# User experience and perceived impact of the Eating Disorder Support App: a mixed-methods evaluation

**DOI:** 10.1186/s40337-026-01655-1

**Published:** 2026-06-24

**Authors:** Veronika Rysinova, Christine E. Ramsey-Wade, Carolyn Trippick, Sanni Norweg

**Affiliations:** 1 School of Social Sciences, University of the West of England, Bristol, UK; 2 Centre for Appearance Research, University of the West of England, Bristol, UK; 3 Eating Disorders Health Integration Team (EDHIT), Bristol Health Partners, Bristol, UK; 4 Avon and Wiltshire Mental Health Partnership NHS Trust, Bristol, UK

**Keywords:** Eating disorders, mHealth, Digital intervention, Mobile application, Lived experience, Co-production, Mixed-methods, App evaluation

## Abstract

**Background:**

Mobile health (mHealth) interventions offer opportunities to improve access to safe, evidence-informed support for people affected by eating disorders (EDs). However, challenges remain around sustained engagement, inclusivity, and integration with care. The co-produced Eating Disorder Support App, developed by the Eating Disorder Health Integration Team (EDHIT) from Bristol Health Partners in the UK, aims to provide trusted, accessible information for individuals with lived experience, supporters, and healthcare professionals. This study examined user engagement, acceptability, and the perceived impact of the app on knowledge, confidence and support-seeking, while identifying opportunities for refinement.

**Methods:**

A mixed-methods evaluation was conducted using an online survey and follow-up semi-structured interviews. Sixteen participants (people with lived experience, supporters, and healthcare professionals) completed a survey assessing app use, usability, satisfaction, and perceived impact; four took part in qualitative interviews exploring experiences in depth. Quantitative data were analysed descriptively, and qualitative data were analysed using conventional qualitative content analysis.

**Results:**

Within this sample, participants reported high satisfaction with the app’s accessibility, clarity, and non-judgemental tone, with the majority (93.8%) rating its overall quality as good or excellent. Survey findings indicated perceived improvements in knowledge, confidence, and intentions to seek or support help-seeking. Qualitative content analysis produced four overarching categories, with ten subcategories, providing further insight into how the app functioned as a trusted, accessible adjunct to care, supporting understanding, reassurance, and emotional safety. Participants also highlighted areas for improvement, including the need for greater personalisation, expanded multimedia content, and clearer navigation to manage initial information load. Overall, findings highlighted the app’s perceived value, while identifying opportunities to enhance engagement and inclusivity.

**Conclusions:**

This evaluation suggests that the Eating Disorder Support App may be an acceptable and useful digital resource that complements clinical care and supports self-management. Future evaluations should include larger and more diverse samples, longitudinal follow-up, and assessment of clinical outcomes to strengthen the understanding of impact. Continued co-production and lived-experience involvement are likely to enhance inclusivity, relevance, and sustained engagement in digital ED support tools.

**Supplementary Information:**

The online version contains supplementary material available at https://doi.org/10.1186/s40337-026-01655-1.

## Abstract (plain language summary)

This study examined how people used and experienced the Eating Disorder Support App, a mobile health tool designed to provide reliable, evidence based information and support for individuals with eating disorders, their families, and healthcare professionals. Sixteen participants used the app and shared feedback through an online survey, with four completing follow-up interviews. The aim of the survey was to understand the user interaction and experience, including what people thought of it, how helpful it felt, and how it could be improved. The interviews gave deeper insights into personal experiences. Findings showed high satisfaction, with users valuing the app’s accessibility, clear layout, trustworthy content, and supportive, non-judgmental tone. Many reported improved knowledge, alongside increased motivation, confidence, and positive behaviour change. Healthcare professionals highlighted its role as a trusted resource to recommend to patients and families. Overall, 93.8% rated the app’s quality as good or excellent. Participants also identified areas for development, including more diverse resources (e.g. content on neurodiversity), alternative formats such as audio or video, and avoiding overwhelming detail. These findings highlight the importance of involving people with lived experience in designing and developing digital health tools. Doing so ensures apps remain engaging, relevant, and effective.

**Supplementary Information:**

The online version contains supplementary material available at https://doi.org/10.1186/s40337-026-01655-1.

## Background

Eating disorders (EDs) are severe mental health conditions associated with high mortality, diminished quality of life, and substantial economic burden [1, 2]. Lifetime prevalence estimates suggest that up to 18.6% of women and 6.5% of men experience a diagnosable eating disorder [3], with increasing incidence over the past 18 years and further escalations observed during the COVID-19 pandemic [4]. International evidence indicates that by early adulthood, between 5.5 and 17.9% of young women and 0.6–2.4% of young men develop an ED [5]. Both prodromal and formally diagnosed EDs are linked with elevated medical and psychological comorbidities, significant functional impairment, and one of the highest mortality rates among psychiatric conditions [6, 7]. The global cost of the disease burden was estimated at US $64.7 billion in 2021 [8].

A range of evidence-based psychological treatments exist, including enhanced cognitive behavioural therapy (CBT-E) [9], the Maudsley Model of Anorexia Nervosa Treatment for Adults (MANTRA) [10], focal psychodynamic therapy (FPT) [11], mentalisation-based treatment [12], pharmacotherapy [13, 14], and family-based treatment for children and adolescents [15]. Despite this, fewer than a quarter of those with, or at risk of, an eating disorder seek or receive support [16–18]. Common barriers include limited specialist availability, financial constraints, long waiting lists, geographical access challenges, concerns about privacy, fear of change and the stigma associated with help-seeking [19–21].

Digital and mobile-health (mHealth) approaches have emerged as promising strategies to reduce barriers to access [22]. Increased global smartphone use has fuelled a rapid expansion of digital health tools [23, 24], which offer opportunities to access psychoeducation, self-help, and treatment resources flexibly, confidentially, and at low cost [25, 26]. Evidence supports the acceptability, effectiveness, and cost-effectiveness of mHealth interventions for common mental health conditions [17], as well as emerging promise for eating disorders [27]. Web-based self-help appears moderately acceptable and accessible [28], with some evidence of comparable effectiveness to in-person interventions [27, 29]. However, mHealth interventions frequently face challenges related to low uptake, limited engagement, and high attrition [30]. Recent evidence suggests that 41% of participants in mental-health app trials do not download the prescribed app, and only one-third complete all modules [31]. For individuals with lived experience of EDs, low engagement may reflect ambivalence towards change [20] and concerns about privacy or content quality [32]. High attrition and low sustained engagement therefore remain significant challenges in the digital ED space [27, 33].

Recognising these limitations and the wide availability of misleading or harmful ED-related information online [34, 35], the Eating Disorder Health Integration Team (EDHIT) from Bristol Health Partners in the UK developed the Eating Disorder Support App. The app aims to provide reliable, evidence-informed, and easily accessible information and resources for individuals with lived experience, carers, and healthcare professionals. Importantly, the app was co-produced, addressing a key gap in the ED app landscape [36]. Previous literature has highlighted that many ED apps are not designed collaboratively with end users [27], do not incorporate lived-experience perspectives, and often fail to follow best-practice standards in user engagement or safety [27, 37]. Participatory research and co-design are therefore recognised as essential for developing acceptable, safe, and engaging digital interventions [38, 39].

The Eating Disorder Support App was developed through a rigorous co-production process involving people with lived experience, carers, specialist clinicians, mental-health practitioners, researchers, and digital designers. The development followed recognised user-centred design principles [40–42], including iterative prototyping, structured feedback cycles, and repeated user testing, consistent with best practice in co-production and Patient Information Forum (PIF) TICK guidance. All content was benchmarked against evidence-based clinical guidelines, reviewed for clarity, safety, and inclusivity, and refined through multiple consultation rounds. The app also obtained external quality assurance via PIF TICK, demonstrating adherence to nationally recognised standards for health information production, including user involvement, accessibility, and impact evaluation. As of April 2026, the app has been downloaded more than 23,700 times, indicating substantial public demand for reliable and accessible eating-disorder information. The ED Support App is currently the only eating disorder mobile app recommended by Mind, a national mental health charity in the UK, and it is listed in its ‘Mind app library’ of accredited and trusted mental health apps: https://mind.orchahealth.com.

Recent studies have emphasised the importance of clinicians’ perspectives in app adoption: 63% of mental health clinicians discuss apps with patients, yet many remain uncertain about which apps are appropriate, safe, and evidence-based [43]. Research also highlights users’ desire for accessible, empowering, personalised, and non-judgmental digital ED resources [44–46], while engagement studies highlight early drop-off in use among individuals seeking support for binge eating [47]. Collectively, these findings highlight the need for ongoing evaluation of real-world mHealth tools developed using best-practice co-production methods.

Given the prevalence of unregulated and potentially harmful ED-related content online, the importance of co-production, and the limited evidence on user experiences of ED-specific apps developed to recognised quality standards, systematic evaluation is essential. This study therefore used a mixed-methods approach to examine user engagement, acceptability, perceived impact, and opportunities for refinement. By integrating insights from people with lived experience, supporters, and healthcare professionals, the evaluation aimed to support the ongoing development and optimisation of safe, acceptable, and evidence-informed digital support tools for eating disorders.

## Methods

### Study design

A mixed-methods evaluation was conducted to explore user experiences and the perceived impact of the Eating Disorder Support App. The study combined an online cross-sectional survey with qualitative semi-structured interviews. This design enabled integration of quantitative indicators of satisfaction and perceived usefulness with in-depth qualitative insights into app experiences [39]. The survey and interview components were designed to be complementary. The survey captured patterns of app use, usability, satisfaction, and perceived impact across the sample, while the qualitative interviews explored these experiences in greater depth and provided contextual insight into the quantitative findings. The evaluation was informed by a pragmatic approach appropriate to applied service and product evaluation [48].

### Setting and intervention

The Eating Disorder Support App was co-produced by the Eating Disorder Health Integration Team (EDHIT) and Expert Self Care Ltd., with sustained involvement from people with lived experience, carers, and clinicians. The app provides evidence-informed, information-based support including psychoeducation, signposting to services, recovery and wellbeing content, and resources for families and healthcare professionals. The app aimed to provide accessible support and care navigation for individuals affected by eating disorders, functioning as a supportive adjunct to care rather than replacing therapy or clinical input. Content development was informed by established behavioural and social models (Health Belief Model [49]; Transtheoretical Model/Stages of Change [50]; Social Cognitive Theory [51]; Social Ecological Model [52]) and underwent iterative user testing, participant group workshops and clinical review. The app meets national health information quality standards and holds the PIF TICK quality mark. As of April 2026, the app has been downloaded over 23,700 times across iOS and Android, with over 64,700 content pageviews (averaging approximately 1,176 per month), suggesting sustained active use beyond initial download. It is the only eating disorder app recommended by the national British mental health charity Mind.

**Fig. 1:**
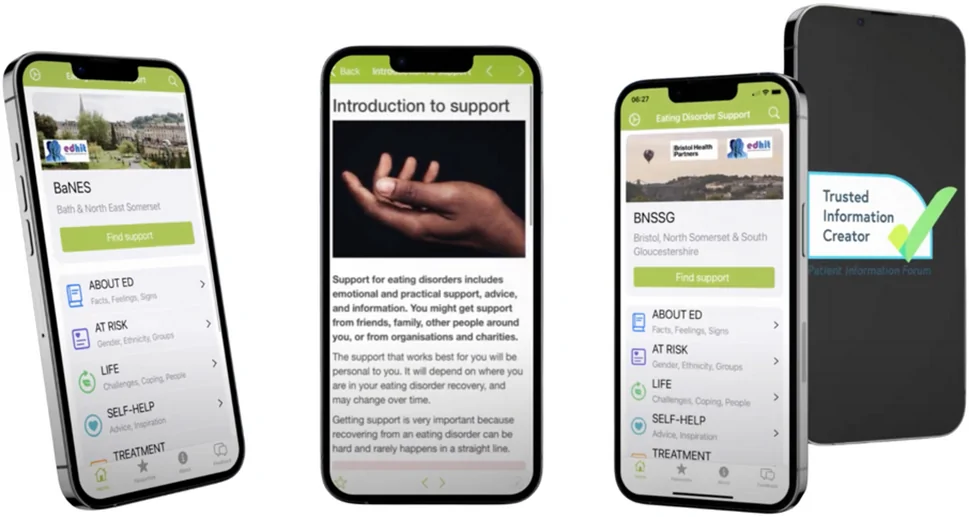
The eating disorder support app

The app does not require user registration and does not collect personal data or granular usage analytics. This absence is a deliberate feature of the app, explicitly prioritised in co-design with people with lived experience, who requested this to protect user confidentiality and lower the barrier to access. This means we are unable to provide further engagement metrics beyond total download figures (Fig. 1).

### Participants

An opportunity sample of 16 survey respondents was recruited. No minimum level of app engagement was required for survey participation. Participants were eligible if they were: aged 18 years or over; able to understand English; and had used or engaged with the app at least once. This approach was intended to capture a range of user experiences, including both initial and more sustained engagement. Key participant groups included: (a) individuals with lived experience of an eating disorder or disordered eating; (b) supporters (family, friends, or carers); and (c) healthcare professionals (HCPs). Participants’ ages ranged from < 25 to 55–64 years. The sample included 14 women and one participant who did not disclose gender (one response was missing/withheld).

The survey was sent out between January–March 2024 via the EDHIT newsletter, social-media posts (Instagram and X), community partners, and an in-app invitation. To support engagement, respondents could enter a random draw for a £20 Asda voucher. A subset of four volunteers (two with lived experience and two HCPs) subsequently completed qualitative interviews.

The adequacy of the qualitative interview sample was considered in relation to information power [53]. Given the specific study aim, the purposive and homogeneous sample, the applied descriptive aims of conventional qualitative content analysis, and the substantive interview accounts obtained, a sample of four participants was considered to carry sufficient information power for the purposes of this evaluation. Findings should nonetheless be treated as exploratory, and transferability to other user groups and contexts is limited, as discussed in the Limitations.

## Measures and materials

### Survey

A bespoke Qualtrics survey was developed to explore participants’ demographic characteristics, participant role, and patterns of app use, including frequency, sections accessed, and duration of engagement. The survey also assessed usability, accessibility, and satisfaction using Likert-scale items, and examined perceived impacts on knowledge, confidence, help-seeking, and wellbeing. In addition, participants were invited to comment on inclusivity, tone, and areas for improvement through open-text responses.

Survey development was informed by established mHealth and app-evaluation frameworks, including the System Usability Scale (SUS) [54], the Mobile Application Rating Scale (MARS) [55], and the Client Satisfaction Questionnaire-8 (CSQ-8) [56], which have been applied in previous evaluations of eating-disorder–related digital tools [57–59]. As no single validated instrument specific to ED mHealth addressed the full scope of our evaluation aims, items were adapted to reflect the target population and the informational nature of the app. Item pools and wording were refined in collaboration with Patient and Public Involvement and Engagement (PPIE) advisors and the clinical team to enhance clarity, relevance, and acceptability. The full survey instrument is provided in Additional File 1.

### Qualitative interviews

Nine survey participants expressed interest in taking part in a qualitative interview; of these, four responded to the invitation and were able to participate. Semi-structured interviews explored first impressions, perceived usefulness/impact, tone and inclusivity, accessibility/usability, and enhancement priorities. Questions were co-developed with PPIE contributors and clinicians, who contributed expert input to both the survey questionnaire and interview schedules. Interviews were conducted via Microsoft Teams, audio-recorded with consent, and transcribed verbatim. Quantitative analysis used IBM SPSS Statistics v29. Qualitative data management and coding used NVivo (release 1.5). The full interview schedule is provided in Additional File 1.

### Procedure

Survey adverts directed potential participants to the study information and consent page hosted in Qualtrics. After e-consent, participants completed the 10–15-minute survey. At the end, they could volunteer for an interview by submitting contact details through a separate, unlinked form (maintaining anonymity of survey responses). Interview volunteers were contacted to arrange a Teams appointment; interviews lasted from 26 min to 62 min (mean approximately 47 min). All participants were informed that participation was voluntary, data would be anonymised, and they could withdraw without giving a reason up to the point of data analysis.

### Ethical considerations

This evaluation was conducted under the governance of the Eating Disorder Health Integration Team (EDHIT) within the Bristol, North Somerset and South Gloucestershire region. According to the Health Research Authority (HRA) decision tool, it was classified as a service or product evaluation and therefore did not require formal research ethics committee review. Procedures adhered to the British Psychological Society (BPS) Code of Human Research Ethics (2014) and the Declaration of Helsinki (1964 and subsequent amendments) [60], and were consistent with Health and Care Professions Council (HCPC) professional standards. Participants received information about the survey purpose, confidentiality, and their right to withdraw, and provided informed electronic consent before participation. Data were stored on secure, encrypted university systems; transcripts were anonymised prior to analysis.

### Data analysis

#### Quantitative

Descriptive statistics (frequencies, percentages, means and standard deviations) were generated for demographic variables, app-use patterns, usability and satisfaction ratings, and perceived impacts. Where useful for interpretation, simple cross-tabulations were conducted to explore variation across participant groups. Extended quantitative results are available in Additional File 1.

#### Qualitative

Interview transcripts were analysed using conventional qualitative content analysis [61]. This approach was selected because the qualitative analysis aimed to systematically summarise and organise participants’ experiences and perspectives in relation to app use, including perceived benefits, usability, emotional impact, and areas for improvement, within an applied evaluation context. Analysis began with repeated reading of the transcripts to support familiarisation with the data. Meaningful segments of text relevant to the evaluation aims were then coded inductively, while remaining attentive to domains identified through the survey findings, including accessibility, usefulness, inclusivity, and perceived impact, in keeping with the convergent mixed methods design. Codes were compared across transcripts and grouped according to similarity in content to form broader categories. These categories were then iteratively reviewed, refined, and organised into four overarching categories, each comprising multiple subcategories, reflecting key patterns across the dataset. The coding framework and verbatim participant extracts are provided in Additional File 1.

### Patient and public involvement and engagement

Lived-experience involvement was embedded throughout. Co-author CT, Peer Co-Director of EDHIT and a person with lived experience of eating disorders, contributed across the full arc of the project, including evaluation design, participant recruitment materials and social media strategy, instrument development, review during analysis, and manuscript review. This reflects a model of co-production in which lived-experience expertise holds an active role at every phase rather than a consultative one [62]. CT’s pre-collection review of the survey instrument led to direct revisions, including corrections to the confidentiality statement, geographical response categories, and inclusive language of survey items and interview schedules.

A broader PPIE group contributed to reviewing and advising on the survey and interview schedule prior to data collection, focusing on accessibility of language and clarity of the participant-facing introduction, providing tracked-change revisions to the interview schedule and refining questions to improve conversational flow and accessibility of phrasing. Finally, following completion of the analysis, preliminary findings and the full evaluation report were shared with participants, who were invited to provide structured feedback on both the findings and the evaluation process via a brief feedback form. This closing stage returned findings to those who had contributed their experiences and created a formal mechanism for accountability, reflecting a model of involvement that extended beyond data collection into active reciprocity with participants.

## Results

### Characteristics of survey respondents

A total of 16 respondents completed the online survey. Of these, seven (43.8%) reported personal experience of an eating disorder, four (25.0%) had experience supporting someone with an eating disorder, and five (31.3%) reported both personal and supportive experience. Participants represented a range of ages (under 25 to 55–64 years) and locations across the United Kingdom (UK), with a limited range of gender identities (93.8% women). (Table 1). Ten participants (62.5%) identified as healthcare professionals.

Four participants subsequently took part in qualitative interviews: two healthcare professionals (a mental-health nurse specialising in eating disorders and a weight-management practitioner) and two individuals with lived experience (Table 2).

The 16 survey respondents and four interview participants represent a purposively recruited subset of a substantially larger user base. The app has been downloaded more than 23,700 times as of April 2026, with over 64,700 content pageviews recorded, indicating considerable real-world reach and sustained active use beyond the initial download. The findings represent a subset of engaged users from a substantially larger download base and should not be read as population-level estimates of experience or satisfaction.

**Table 1:** Demographic and relevant sample characteristics of the survey group of those with lived experience and healthcare professionals; *n* = 16

Demographics	Survey sample *n* = 16		
Age	Under 25 (%)	2 (12.5)	
	25–34 (%)	2 (12.5)	
	35–44 (%)	6 (37.5)	
	45–54 (%)	4 (25.0)	
	54–64 (%)	2 (12.5)	
Gender	Women (%)	15 (93.8)	
	Prefer not to say (%)	1 (6.3)	
Ethnicity	White (%)	10 (62.5)	
	White other (%)	6 (37.5)	
Health professional	Yes (%)	10 (62.5)	
	No (%)	6 (37.5)	
Location (UK)	Bath and North East Somerset (BANES)	2 (12.5)	
	Bristol	3 (18.8)	
	South Gloucestershire	2 (12.5)	
	Other	9 (56.3)	
ED-related experience	I have a personal experience with an eating disorder	7 (43.8)	
	I have supported someone with an eating disorder.	4 (25.0)	
	I have both personal and supportive experiences.	5 (31.3)	

**Table 2:** Interviewees and relevant description

Interview transcript	Description
Participant 1	Mental health nurse specialising in EDs
Participant 2	Weight management practitioner
Participant 3	Person with lived experience
Participant 4	Person with lived experience

### App usage patterns

Patterns of app engagement varied across respondents (Table 3). Most respondents reported using the app rarely (37.5%) or several times per month (25.0%), while one participant reported daily use (6.3%). Half of the sample (50.0%) had used the app for less than one month, whereas one quarter (25.0%) had used it for more than a year.

Participants selected multiple reasons for using the app. The most commonly reported purposes included accessing resources (17.6%), supporting someone with an eating disorder (17.6%), and seeking general information about eating disorders (17.6%). A smaller proportion reported using the app primarily for self-help (14.7%). Other motivations included learning about symptoms, understanding treatment options, and identifying risk factors. Reasons for use were assessed through a multiple-choice format; percentages therefore reflect the proportion of total selections rather than participants.

**Table 3:** App usage profiles of the survey respondents

App usage profile	Survey sample *n* = 16 (%)		
App usage frequency	Daily	1 (6.3)	
	Several times a week	2 (12.5)	
	Once a week	3 (18.8)	
	Several times a month	4 (25.0)	
	Rarely	6 (37.5)	
App usage length	< 1 month	8 (50.0)	
	1–3 months	2 (12.5)	
	3–6 months	1 (6.3)	
	6–12 months	1 (6.3)	
	> 1 year	4 (25.0)	
Reasons for using the app	To gain knowledge about eating disorders	4 (11.8)	
	To understand symptoms and effects	3 (8.8)	
	To find out more about treatment	3 (8.8)	
	To access resources	6 (17.6)	
	To provide support to an individual with lived experience	6 (17.6)	
	For self-help	5 (14.7)	
	To find out who is at risk of getting an eating disorder	1 (2.9)	
	Other	6 (17.6)	

### Exploratory comparison by engagement frequency

Given the variability in quantitative responses across the sample, a descriptive cross-tabulation of app use frequency against key outcome variables was conducted. Findings are exploratory only; no inferential statistics were applied. Respondents were grouped into higher engagement (weekly or more; *n* = 6) and lower engagement (monthly or rarely; *n* = 10).

A consistent directional pattern was observed across most outcomes, with higher-engagement respondents tending to report more positive ratings, including: perceived impact on awareness (mean 4.00 vs. 3.40 on a five-point scale), knowledge increase (4.00 vs. 3.30), interest and engagement (4.00 vs. 2.70), functionality (3.50 vs. 2.80 on a four-point scale), overall quality (3.50 vs. 3.40), and extent to which the app met needs (3.17 [*n* = 6] vs. 2.57 [*n* = 7]; three lower-engagement respondents did not respond to this item, see Table 4). The most pronounced difference was on interest and engagement, where all three “not interesting at all” ratings came from lower-engagement respondents and both “extremely interesting” ratings came from higher-engagement respondents. On functionality, both “poor” and “average” ratings came exclusively from the lower-engagement group. The exception was perceived usefulness of informational content, rated similarly across both groups (higher: mean 4.00, *n* = 5; lower: mean 4.10, *n* = 10), suggesting the app functions as a valued reference resource regardless of engagement frequency.

**Table 4:** Exploratory descriptive comparison of key outcome variable ratings by engagement frequency group (*n* = 16)

Outcome variable	Higher engagement (weekly or more; *n* = 6)		Lower engagement (monthly or rarely; *n* = 10)	
	Mean	*n*	Mean	*n*
Awareness impact	4.00	6	3.40	10
Knowledge increase	4.00	6	3.30	10
Information usefulness	4.00	5	4.10	10
Interest and engagement	4.00	6	2.70	10
Functionality	3.50	6	2.80	10
Overall quality	3.50	6	3.40	10
Needs met	3.17	6	2.57	7

### Overview of categories and subcategories

Four overarching categories (and 10 subcategories) were identified through qualitative content analysis, integrating insights from both survey comments and interview data (Table 5). These were: (1) supporting understanding, confidence and empowerment; (2) navigating and engaging with the app; (3) areas for development and concerns; and (4) creating an inclusive, sensitive and emotionally safe space. The categories and subcategories are summarised in Table 5 and discussed in detail in the sections that follow, alongside relevant quantitative survey findings to contextualise and extend the qualitative findings, building on the engagement patterns described above. While many experiences were shared across individuals with lived experience and healthcare professionals, some accounts were more pronounced within particular participant groups, highlighting both convergence and divergence in perspectives.

**Table 5:** Summary of the categories and subcategories across the data set

Category	Subcategory	Definition
1. Supporting understanding, confidence and empowerment	1.1 Easy access, satisfaction and integration with care	Respondents experienced the app as easy to access and navigate, felt satisfied with its use, and perceived it as a supportive complement to existing ED care or support pathways.
	1.2 Trustworthy information offering something “extra”	Perceptions that the app provides reliable, evidence-informed information that adds value beyond routine clinical support, informal advice or typical online sources.
	1.3 Increased confidence, mindset shifts and behavioural support	Experiences of greater confidence, reassurance, and shifts in thinking or behaviour linked to engaging with the app.
	1.4 Feeling supported, validated and empowered	Accounts of the app fostering feelings of support, emotional validation and empowerment through its tone, content and availability at times of need.
2. Navigating and engaging with the app	2.1 Clear structure, layout and navigation	Views on the organisation, clarity and intuitive navigation of the app, contributing to a smooth user experience.
	2.2 Engagement features, delivery style and interactivity	Reflections on engaging elements (e.g., design, content features, delivery style) and suggestions for strengthened interactivity or visual appeal.
3. Areas for development and concerns	3.1 Desire for personalisation and broader resource options	Suggestions for increased tailoring (e.g., personalised pathways or targeted content) and expansion of the resource base available within the app.
	3.2 Managing information load and expectations	Concerns about information density or complexity, and reflections on the importance of clearly communicating the app’s purpose and limits to avoid overwhelm.
4. Creating an inclusive, sensitive and emotionally safe space	4.1 Inclusivity and sensitivity to diverse experiences	Perceptions of how well the app acknowledges and respects diverse identities, lived experiences and needs.
	4.2 Clear, warm and non-judgemental communication	Views on the clarity, warmth and non-judgemental tone of the language used, contributing to emotional safety and ease of engagement.

### Category 1: supporting understanding, confidence and empowerment

The clearest finding across all participant groups was that the app functioned not primarily as an information repository but as a resource that actively supported users’ sense of agency, confidence and reassurance, and the capacity to navigate eating disorder challenges. Across the four subcategories, the app’s value was understood in relational terms: it helped users feel less alone, better equipped, and more confident in their interactions with the healthcare system and with those they supported.

#### Easy access, satisfaction and integration with care

Accessibility and integration with existing clinical pathways underpinned users’ overall confidence in and satisfaction with the app, and shaped their willingness to recommend it to others. Participants consistently described the app as easy to access, straightforward to use and valuable alongside clinical care. Both individuals with lived experience and healthcare professionals appreciated having reliable, evidence-informed information available immediately:

*“My clients find it useful… having that information readily available is really important.”* (Participant 1) *“You can access it whenever you need it.”* (Participant 3).

Healthcare professionals noted that the app fit well with treatment pathways and acted as consistent support between appointments: *“It crosses over nicely with our treatment pathways.”* (Participant 1).

Survey findings aligned strongly: 93.8% rated the app as good or excellent, 75% said it met their information needs, and 87.5% would recommend it to someone experiencing eating difficulties (Table 6). 75% reported they would use the app again. One participant summarised: *“A very innovative idea that could help a lot of people… providing carers or loved ones with information and easy signposting.”* (Survey).

**Table 6:** App satisfaction ratings and percentages of the survey participants

App satisfaction	Survey sample *n* = 16 (%)		
How would you rate the quality of the app?	Excellent	8 (50.0)	
	Good	7 (43.8)	
	Fair	1 (6.3)	
	Poor	0	
	Very poor	0	
Did the app offer the type of support/information you were looking for?	Not at all	1 (6.3)	
	Not particularly	1 (6.3)	
	I’m not sure	2 (12.5)	
	Yes, generally	7 (43.8)	
	Yes, definitely	5 (31.3)	
To what extent has the app met your needs?	Almost all of my needs have been met	5 (31.3)	
	Most of my needs have been met	3 (18.8)	
	Only a few of my needs have been met	3 (18.8)	
	None of my needs have been met	2 (12.5)	
If a friend was dealing with eating disorders, would you recommend this app?	Not at all	0	
	Not particularly	1 (6.3)	
	I’m not sure	1 (6.3)	
	Yes, I think so	5 (31.3)	
	Yes, definitely	9 (56.3)	
If you were to seek help again, would you choose this app?	Not at all	0	
	Not particularly	2 (12.5)	
	I’m not sure	2 (12.5)	
	Yes, I think so	7 (43.8)	
	Yes, definitely	5 (31.3)	

#### Trustworthy information offering something “extra”

A consistent pattern across interview and survey data was that the app’s value derived not simply from its informational content but from the trust participants placed in that content and the relief that trust provided. Participants described the app as filling a meaningful gap by offering credible, well-organised information that contrasted sharply with the experience of searching online.

*“The information is factual… they don’t have to go searching for it.”* (Participant 1); *You don’t have to panic-Google… sometimes you go down a rabbit hole.”* (Participant 4).

Healthcare professionals viewed the app as a meaningful enhancement to their practice:

*“A really nice resource… something extra I can advise people to look at.”* (Participant 1).

The thoroughness of the content also contributed to trust: *“It looks very genuine because it’s so comprehensive… effort has been put into this app.”* (Participant 3).

Survey responses strongly supported these views: 62.5% found the information very or extremely useful, 75% rated it as accurate and reliable, and 93.8% as mostly or highly relevant. Only one participant noted that some content felt less applicable to a longstanding illness (Table 7).

**Table 7:** Participants’ ratings regarding the information usefulness, accuracy, reliability, relevance, and comprehensiveness

App information	Survey sample *n* = 16 (%)		
How useful is the information about eating disorders in the app?	Not at all useful	0	
	Slightly useful	0	
	Moderately useful	5 (31.3)	
	Very useful	4 (25.0)	
	Extremely useful	6 (37.5)	
How accurate and reliable is the information presented in the app?	Very inaccurate/unreliable	1 (6.3)	
	Inaccurate/unreliable	0	
	I don’t know	3 (18.8)	
	Accurate/reliable	6 (37.5)	
	Very accurate/reliable	6 (37.5)	
How relevant is the information on the app to eating disorders?	Not relevant at all	0	
	Slightly relevant	0	
	Neutral	1 (6.3)	
	Mostly relevant	5 (31.3)	
	Highly relevant	10 (62.5)	
How would you rate the comprehensiveness of the provided information?	Not comprehensive at all	0	
	Slightly comprehensive	0	
	Moderately comprehensive	6 (37.5)	
	Very comprehensive	5 (31.3)	
	Extremely comprehensive	5 (31.3)	

#### Increased confidence, mindset shifts and behavioural support

Beyond informational value, participants described the app as contributing to meaningful shifts in confidence, motivation, and emotional outlook. For some, this translated into tangible behavioural change. Participants described increases in confidence, motivation and emotional security following app use. For some, the app reduced anxiety in social contexts and encouraged re-engagement in activities they previously avoided: *“It’s given me motivation… made me more confident to engage in situations I wouldn’t have before.”* (Participant 3) The app also prompted reflection on relationships, boundaries and wellbeing: *“It made me think about people I no longer engaged with… it forced you to look at maintaining good relationships.”* (Participant 3).

Self-help materials, journaling tools and emotional-expression resources were described as especially valuable: *“Journaling… really helped with the management of it.”* (Participant 3).

Healthcare professionals also drew on the app to support discussions about thinking styles, realistic goals and symptom recognition: *“The information about unhelpful thinking styles… they found that really helpful.”* (Participant 1).

Survey findings strongly reflected the positive qualitative accounts. Most respondents reported that the app increased their awareness of eating disorders (62.5%), enhanced their knowledge (62.5%), and was likely to increase their motivation to address or overcome disordered eating (81.3%). Many anticipated that the app would encourage help-seeking (87.5%) and contribute to positive behaviour change (68.8%), with only a small minority perceiving little impact (Table 8).

**Table 8:** Participants’ ratings regarding the impact of the app on awareness, knowledge, motivation, help-seeking, and behaviour change

App impact	Survey sample *n* = 16 (%)		
How impactful has the app been in raising your awareness about eating disorders?	Very impactful	4 (25.0)	
	Moderately impactful	6 (37.5)	
	Neutral	4 (25.0)	
	Slightly impactful	0	
	Not at all impactful	2 (12.5)	
How effective has the app been in increasing your knowledge/understanding of eating disorders?	Very effective	4 (25.0)	
	Moderately effective	5 (31.3)	
	Neutral	5 (31.3)	
	Slightly effective	0	
	Not effective	2 (12.5)	
How likely do you think the use of this app is to increase intentions/motivation to address/overcome disordered eating habits?	Highly likely	3 (18.8)	
	Likely	10 (62.5)	
	Neutral	1 (6.3)	
	Unlikely	2 (12.5)	
	Highly Unlikely	0	
How likely do you think the use of this app is to encourage further help seeing for individuals dealing with eating disorders?	Highly likely	5 (31.3)	
	Likely	9 (56.3)	
	Neutral	2 (12.5)	
	Unlikely	0	
	Highly Unlikely	0	
How likely do you the use of this app is to contribute to positive behaviour changes in managing and overcoming disordered eating?	Highly likely	1 (6.3)	
	Likely	10 (62.5)	
	Neutral	4 (25.0)	
	Unlikely	1 (6.3)	
	Highly Unlikely	0	

#### Feeling supported, validated and empowered

Perhaps the most consistent finding across participant groups was that the app provided something beyond information - a sense of ongoing presence, safety, and validation that persisted even when professional support was not immediately accessible. This was particularly valued during the gaps that characterise eating disorder care pathways.

*“It’s something I signpost clients to… peace of mind for me as well.”* (Participant 2) Individuals with lived experience described the app as a secure, private companion: *“You always need that security… having this on your phone is different.”* (Participant 3) *“There’s a sense of security… it’s not going to disappear.”* (Participant 3) Participants also described feeling more validated, less dismissed and more positive: *“It made me more positive… you wouldn’t feel dismissed.”* (Participant 3).

Survey findings reinforced these qualitative insights. Most respondents believed the app made them feel more equipped or knowledgeable about supporting someone with an eating disorder (81.3%), and many felt it helped them deal more effectively with related challenges (43.8%). Importantly, none of the respondents reported that the app made things worse (Table 9). Participants also described the app as reassuring: “*It provides me with a sense of security”* (Survey) and valued its supportive tone and resources for both themselves and loved ones.

**Table 9:** Participants’ ratings regarding the impact of the app on feeling equipped to support or deal more effectively with related challenges

App support	Survey sample *n* = 16 (%)		
How likely do you think the use of this app is to contribute to feeling more equipped or knowledgeable about supporting someone with an eating disorder?	Highly likely	8 (50.0)	
	Likely	5 (31.3)	
	Neutral	2 (12.5)	
	Unlikely	1 (6.3)	
	Highly Unlikely	0	
Has the app helped you to deal more effectively with related challenges?	Yes, it helped a great deal	5 (31.3)	
	Yes, it helped somewhat	2 (12.5)	
	No, it really didn’t help	2 (12.5)	
	No, it seemed to make things worse	0	

### Category 2: navigating and engaging with the app

Across this sample, the app’s structural clarity and ease of navigation were experienced as functional prerequisites for engagement, enabling users to access what they needed, when they needed it, without adding to their cognitive load. This was particularly valued during moments of distress. At the same time, a subset of participants identified the interface design as a limitation, pointing to a tension between comprehensive content and the interactivity conventions of contemporary app design.

#### Clear structure, layout and navigation

Within this sample, the app’s structural organisation was experienced as a core enabler of use, particularly during moments of acute distress when navigational clarity reduced the barrier to accessing support. Participants consistently described the app’s structure and layout as clear, well-organised and easy to navigate. The ability to quickly locate relevant information, particularly during moments of distress, was viewed as highly important:

*“If you’re looking for crisis stuff*,* you can find that in two buttons… it’s structured really well and easy to read.”* (Participant 1).

Users appreciated the organisation of sections and subsections, which improved clarity and reduced cognitive load: *“It was very well laid out… you were able to click into what you thought you needed.”* (Participant 3). Features such as colour-coded highlights, bullet points and recognisable icons supported navigation and readability: *“I like that important information is flagged… the colours break up the text.”* (Participant 2) *“The icons were good—exactly what people would associate with those things.”* (Participant 4).

Survey findings largely mirrored these accounts (see Table 10). Most respondents rated the navigation as *mostly* or *extremely intuitive* (87.5%) and found the visual design appealing (62.5%). Notably, lower-engagement respondents were more likely to rate functionality negatively, with both “poor” and “average” ratings coming exclusively from this group (Table 4). However, a small number found the layout less intuitive or visually engaging:

*“It was more like an ebook than an app… not very well spaced out… not visually appealing.”* (Survey).

**Table 10:** Participants’ ratings regarding the app’s navigation and visual appeal

App navigation and appeal	Survey sample *n* = 16 (%)		
How intuitive is the app’s navigation and screen changes?	Very confusing	0	
	Somewhat confusing	1 (6.3)	
	Neutral	1 (6.3)	
	Mostly intuitive	10 (62.5)	
	Extremely intuitive	4 (25.0)	
How visually appealing do you find the app?	Not appealing at all	1 (6.3)	
	Slightly appealing	2 (12.5)	
	Moderately appealing	3 (18.8)	
	Very appealing	5 (31.3)	
	Extremely appealing	5 (31.3)	

#### Engagement features, delivery style and interactivity

While participants valued the breadth and relevance of the app’s content, a recurring pattern was the contrast between that content value and the perceived limitations of the interface design. This discrepancy between what the app offered and how it delivered it seemed to be most prominent among users accustomed to more interactive digital formats. Participants valued the app’s breadth of content and the range of features designed to support varying needs. Many emphasised its relevance for younger users and those accustomed to digital formats: *“Apps are proving very useful*,* particularly for our younger clients.”* (Participant 1) Several participants appreciated being able to “check in” with the app regularly, noting that its structure supported both brief use and deeper engagement: *“There are a lot of sections… I do check in on it*,* and I know it’s always there.”* (Participant 3).

The self-help section was consistently described as especially helpful, with content closely aligned with clinical practice: *“The self-help stuff is particularly useful… especially Overcoming Binge Eating*,* which we use in clinic.”* (Participant 1) Participants valued the app’s guided ideas for self-care, managing overwhelming thoughts and supporting emotional regulation: *“There’s a whole section on what to do if you’re struggling… knowing I can check that is really useful.”* (Participant 4) However, some felt that the interface could be updated to enhance engagement: “*The user interface is quite outdated… not very person-friendly.”* (Participant 4).

Survey data reflected a similar spread of views (see Table 11). While many respondents found the app useful for identifying risk factors and signposting clients, others reported limited engagement: *“Great for understanding risk and red flags… lots of links for signposting.”* (Survey); *“Didn’t work to engage me.”* (Survey). Ratings of interest and engagement varied widely, from *not interesting at all* (18.8%) to *extremely interesting* (12.5%), and showed the largest difference between engagement groups in the descriptive comparison (Table 4). Overall functionality was rated positively, with 81.3% describing it as *good* or *excellent*.

**Table 11:** Participants’ ratings regarding the impact of the app engagement, functionality and specific app’s features

App engagement and features	Survey sample *n* = 16 (%)		
How interesting is the app in capturing and maintaining your interest?	Not interesting at all	3 (18.8)	
	Slightly interesting	1 (6.3)	
	Moderately interesting	4 (25.0)	
	Very interesting	6 (37.5)	
	Extremely interesting	2 (12.5)	
Overall, how would you rate the functionality of the app (i.e., how well it does what is supposed to)?	Very poor	0	
	Poor	1 (6.3)	
	Average	2 (12.5)	
	Good	8 (50.0)	
	Excellent	5 (31.3)	
Which area(s) of the app do you find most useful?	Local page	3 (4.3)	
	About ED	7 (10.0)	
	At risk	5 (7.1)	
	Life	6 (8.6)	
	Self help	12 (17.1)	
	Treatment	6 (8.6)	
	Support	10 (14.3)	
	Calm zone	9 (12.9)	
	Supporting others	8 (11.4)	
	Partners	3 (4.3)	
	Other	1 (1.4)	

### Category 3: areas for development and concerns

Participants identified opportunities to strengthen the app, particularly through greater personalisation, broader and more diverse resources, and enhanced accessibility features. While the comprehensive content was appreciated, some users described initial information overload, highlighting the need for clearer signposting and realistic expectations about the app’s role.

#### Desire for personalisation and broader resource options

Across participant groups, the desire for greater personalisation reflected a shared recognition that eating disorder experiences are heterogeneous and that a single fixed content structure cannot fully serve all users. While participants valued the existing resources, many felt that additional tailoring could better support different learning preferences, clinical pathways and cultural contexts. Healthcare professionals noted that although regional information was helpful, expanding national resources would improve relevance across locations: *“The wider resources are really useful… but the resources aren’t national yet.”* (Participant 1).

Users also highlighted the benefit of geographical tailoring, explaining that people often want information specific to their own country or local services: *“Even just seeing to people in your own country… what’s available in your country.”* (Participant 3) Participants suggested integrating more varied media formats to accommodate different learning styles and enhance engagement. Audio options, videos and podcasts were frequently mentioned as ways to support accessibility: *“Videos and podcasts… some people learn better or understand information differently.”* (Participant 2) Another consistent suggestion was for more content focused on maintenance of wellbeing rather than crisis alone, acknowledging that eating disorder recovery is often cyclical: *“Resources around maintaining your wellness… because with an eating disorder you will relapse and have periods of wellness.”* (Participant 4).

Survey data reinforced these needs. Respondents requested content on interoception, neurodiversity, ADHD, menopause-related eating changes, and support for those beyond initial assessment stages. They also emphasised a desire for enhanced accessibility, including audio descriptions and customisable colour schemes, as well as more visual elements and built-in reflective activities rather than relying solely on external links:

*“More images… a space to journal or share thoughts… less wordy.”* (Survey).

*“Provide an audio description… different colour schemes.”* (Survey).

*“More downloadable resources… activities built into the app would be useful.”* (Survey).

#### Managing information load and expectations

This subcategory captures participants’ reflections on the balance between comprehensive information and potential overwhelm, highlighting the importance of managing expectations around the app’s purpose while ensuring clarity and emotional safety for users.

Participants described mixed initial reactions to the volume of information within the app. While many eventually appreciated its comprehensiveness, some reported feeling overwhelmed when first opening it. One participant explained: *“When I first downloaded the app*,* I was a little bit overwhelmed… there was so much information. Over time it became a positive because it’s very comprehensive.”* (Participant 3) Others noted that the density of content could feel confusing, particularly if someone was unsure what they were looking for or using the app during distress: *“It’s a very busy app… I can imagine if you weren’t quite sure what you were accessing*,* it could be confusing.”* (Participant 4).

Participants emphasised that the app itself was not harmful, but unrealistic expectations of what it could deliver might lead to disappointment. As one person reflected: *“It’s done me no harm… the only harm would be if I thought this app would do everything. If I unrealistically looked at it as the be-all and end-all.”* (Participant 3).

Survey responses largely echoed these experiences. While one respondent reported feeling overwhelmed by the amount of information, others praised the app’s clarity and balance:

*“Overwhelmed by information.”* (Survey).

*“Informative without being too overwhelming or wordy.”* (Survey).

*“Good balance between being informative but not overwhelming.”* (Survey).

Quantitative findings showed that the majority of users (68.8%) were “not concerned at all” about the information provided, suggesting that although a minority initially experienced overload, most felt the app maintained an appropriate level of detail and clarity (see Table 12).

**Table 12:** Participants’ ratings regarding concerns about the app

App concerns	Survey sample *n* = 16 (%)		
Do you have any concerns about the information provided by the app?	Highly concerned	0	
	Concerned	0	
	Neutral	3 (18.8)	
	Not very concerned	2 (12.5)	
	Not concerned at all	11 (68.8)	

### Category 4: creating an inclusive, sensitive and emotionally safe space

Respondents viewed the app as inclusive, sensitive and emotionally safe, noting that it addressed diverse identities and experiences often overlooked in eating-disorder resources. The language was consistently described as clear, accessible and non-judgemental, offering reassurance without medical jargon or patronising tones. The convergence of qualitative and survey findings in this category was the strongest across the analysis.

#### Inclusivity and sensitivity to diverse experiences

Within this sample, participants’ accounts of the app’s inclusivity revealed something important about what safe digital engagement requires for people affected by eating disorders: the sense that their particular experience had been considered and represented. They valued that the content acknowledged groups often overlooked in eating-disorder resources, including ethnic minorities, LGBTQ+ individuals, pregnant people, men and people with disabilities. This breadth was viewed as thoughtful, intentional and reflective of real-world diversity:

*“There are sections on all the things we might come across… different ethnic minorities*,* LGBTQI*,* dancers*,* men*,* pregnant people. Although they’re minorities*,* the information is there*,* which is very inclusive.”* (Participant 1).

Participants also felt the app addressed areas commonly omitted in clinical conversations, describing the content as “genuine” and recognising experiences that are *“so often left out”* (Participant 3). This inclusivity contributed to trust and emotional safety, with some users expressing hope that the app would continue to involve diverse communities in its development and feedback processes: *“It covers different cultures… it’d be great if you were getting feedback from those groups. It definitely doesn’t discriminate.”* (Participant 4).

*“It comes from specialists*,* but also from a community of people with lived experience… people have had a voice.”* (Participant 4).

Survey findings reinforced these perceptions. Respondents noted the inclusion of neurodiversity, ADHD and interoceptive impairment as important areas of representation, while a small number raised questions about language accessibility, such as availability in additional languages. Overall, 87.5% rated the app as moderately to extremely inclusive and sensitive (Table 13).

**Table 13:** Participants’ ratings regarding sensitivity, inclusivity, and diversity

App inclusivity	Survey sample *n* = 16 (%)		
How sensitive is the information presented to the needs and concerns of individuals with eating disorders?	Not sensitive at all	0	
	Slightly sensitive	0	
	Moderately sensitive	2 (12.5)	
	Very sensitive	8 (50.0)	
	Extremely sensitive	6 (37.5)	
To what extent do you feel the app includes a diverse range of perspectives and experiences about eating disorders?	Not at all inclusive of diverse perspectives and experiences	0	
	Slightly inclusive of diverse perspectives and experiences	2 (12.5)	
	Moderately inclusive of diverse perspectives and experiences	6 (37.5)	
	Very inclusive of diverse perspectives and experiences	5 (31.3)	
	Extremely inclusive of diverse perspectives and experiences	3 (18.8)	

#### Clear, warm and non-judgemental communication

The app’s communicative tone was consistently described as one of its most important features. It was experienced as informative, but most importantly it was experienced as safe. For participants who had previous experience of feeling judged or dismissed in healthcare contexts, the non-judgemental quality of the language carried particular weight.

*“It’s easy to understand… there’s no jargon. It’s not going to alienate people who aren’t familiar with medical terms.”* (Participant 3).

Many contrasted the app’s tone with some previous experiences of feeling dismissed or blamed in healthcare settings. The app’s factual yet sensitive communication style was described as validating and respectful: *“The tone is not judgmental… you don’t feel as if you’re being given out to. It’s very inclusive.”* (Participant 3). Respondents appreciated that the tone struck a balance, neither overly clinical nor overly informal, providing information in a way that felt appropriate, steadying and emotionally attuned: *“It’s not condescending*,* it’s not too medical… it tells you exactly what you need to know*,* and it hits the tone so well.”* (Participant 4).

Survey responses strongly echoed these impressions (see Table 14). Users described the language as “neutral and informative,” “professional but caring,” and “factual yet comforting.” Others emphasised its accessibility, describing it as “inclusive,” “understandable without being patronising,” and “supportive and non-judgemental.” Most respondents (68.8%) rated the language as *very clear and easy to understand*, and none described it as unclear or difficult.

**Table 14:** Participants ratings regarding the language used in the app

App language	Survey sample *n* = 16 (%)		
How would you rate the understandability of the language used in the app?	Very clear and easy to understand	11 (68.8)	
	Clear and mostly understandable	4 (25.0)	
	Neither clear nor unclear	1 (6.3)	
	Somewhat unclear and challenging to understand	0	
	Very unclear and difficult to understand	0	

## Discussion

### Summary of findings

This evaluation provides exploratory insight into how a small subset of people with lived experience, supporters and healthcare professionals engaged with a co-produced, evidence-informed mHealth app for eating disorders. Within this sample, participants consistently described the app as accessible, trustworthy and easy to navigate, reporting high satisfaction with its clarity, organisation and alignment with existing care pathways. Survey data supported these accounts, with 93.8% respondents rating the app as high quality, relevant and useful. Across groups, respondents reported positive impacts on confidence, understanding and emotional reassurance. Across groups, respondents described the app as a supportive resource between appointments, noting that it encouraged feelings of safety, empowerment and validation. Within this sample, the app’s language was widely experienced as clear, warm and non-judgemental, and its inclusive representation of diverse identities and experiences was viewed as a key strength.

Respondents also identified opportunities for improvement, including greater personalisation, expanded content relating to neurodiversity, interoception and menopause, additional multimedia formats (e.g., audio, video), and clearer signposting to help users manage initial information overload. A small number of respondents noted that some content felt less relevant to long-standing illness or later stages of recovery. These preliminary findings suggest that a co-produced, quality-assured mHealth tool may offer meaningful support to individuals affected by eating disorders, while highlighting areas for continued refinement to enhance relevance, personalisation and long-term engagement, and the challenges of meeting the needs and preferences of all users. Lived experience input guided the design of the app in an e-book style, with no registration requirements and no gathering of personal information, to keep the app simple and usage confidential; these app features currently preclude greater personalisation. Since the completion of this evaluation, a link to a tested, video-based mindful eating micro-intervention has been embedded in the app in response to requests for more accessible and varied information formats.

### Integrative synthesis

Considered together, the quantitative and qualitative findings tell a coherent story about how participants within this sample positioned the app within their broader support system. The overarching pattern, which was consistent across all four categories and the survey data, was that the app’s value was relational as much as informational. It was experienced not as a standalone intervention but as a trust-building, orientation-providing resource that helped users feel less alone, better equipped to navigate the healthcare system, and more confident in conversations with clinicians and family members. The high satisfaction ratings are consistent with and contextualised by the qualitative finding that participants valued not only the informational content but the non-judgemental, emotionally safe character of the app, suggesting that the co-produced design process produced an affective quality of user experience that quantitative data alone could not fully capture.

The exploratory engagement comparison (Table 4) added a further dimension. Higher-engagement respondents tended to rate the app more positively across most outcomes, though the exception (informational usefulness) rated similarly regardless of engagement frequency, points to the app’s value as a reference resource accessible at key moments for respondents. The tension identified in Category 3 between comprehensive content and the risk of information overwhelm reflects a broader challenge in digital mental health design [63]. The features users value most may simultaneously become barriers for those accessing the app during periods of acute distress. Participants’ own framing of this ‘as a risk of unrealistic expectations rather than a flaw in the app’ shows the importance of expectation management as a potentially important component of implementation.

### Comparison with existing literature

The evaluation findings align closely with existing literature on digital mental-health and eating-disorder interventions. Within this sample, respondents’ emphasis on accessibility, clarity and ease of navigation reflects a broader evidence base demonstrating that user-centred, intuitive design is essential for engagement and acceptability in mHealth tools [64–66]. Prior work similarly highlights the importance of simple layouts, clear signposting and minimal cognitive burden, particularly during periods of distress, when sustained engagement is most fragile [57, 67]. The strong sense of safety and trustworthiness reported by participants directly addresses well-established concerns about the prevalence of misleading, unregulated or harmful ED-related information online [35, 68]. This is consistent with reviews demonstrating that many existing ED apps lack clinical oversight, are not co-produced with people with lived experience, and show considerable variability in informational accuracy and quality [37, 59]. In contrast, the co-produced and quality-assured model underpinning the present app appears to mitigate these risks and contribute to positive user perceptions of credibility and reliability, consistent with broader evidence on the value of participatory design in digital health [69].

Participants’ appreciation of the app’s non-judgemental, warm and inclusive tone aligns with evidence that tone, empathy and stigma-sensitive language are central to building digital therapeutic alliance and supporting engagement in mental-health apps [36, 70, 71]. The value placed on diverse representation, including content relevant to different identities, stages of life and cultural contexts, mirrors calls in ED research for more inclusive, intersectional digital resources that reflect the heterogeneity of people affected by eating disorders [72].

The finding that participants experienced the app as a supportive resource between appointments is consistent with literature highlighting the role of digital tools in bridging gaps caused by limited specialist provision, long waiting lists and geographical barriers to care [19–21]. While clinical outcomes were not evaluated in this study, previous research suggests that digital psychoeducation and self-support tools may promote early help-seeking, improve confidence in symptom management, and enhance treatment engagement [73, 74].

Participants’ desire for greater personalisation echoes longstanding evidence that adaptive and tailored digital interventions are more likely to maintain relevance and improve engagement over time [75, 76]. Requests for expanded content relating to neurodiversity, interoception and menopause-related changes align with emerging ED literature that highlights the importance of attending to these specific experiences [77–79]. Likewise, preferences for multimedia formats (e.g., audio, video) are consistent with studies showing that multimodal delivery supports different learning preferences and improves accessibility for users with cognitive or attentional challenges [80, 81]. Finally, mixed experiences related to information overwhelm parallel findings on cognitive load in digital interventions, where initial overload is common and can be mitigated through clearer orientation, pacing and expectation management [82, 83]. This reinforces the need for carefully structured content and personalised pathways, particularly in early stages of engagement.

### Future evaluations

Future studies should examine how engagement, perceived usefulness and behavioural impacts evolve over time, particularly given the natural fluctuations and relapse–recovery cycles that characterise many eating-disorder experiences [84]. Longitudinal designs would help clarify whether the initial benefits reported here are sustained and how users draw on the app during different phases of their recovery journey. Evaluating clinical outcomes, such as help-seeking, treatment adherence, emotional regulation and symptom trajectories, would further strengthen understanding of the app’s effectiveness and its potential role within treatment pathways. Future evaluations should also examine engagement frequency and user role as moderating variables, incorporating objective engagement metrics alongside self-report data to build on the exploratory patterns observed here. Recruiting more diverse samples is essential to improve cultural relevance and ensure the app meets the needs of populations disproportionately affected by structural barriers to ED care. Research exploring implementation within real-world services, including blended-care and stepped-care models, would also provide insight into feasibility, sustainability and optimal integration with existing clinical systems. Future work could also explore adaptive or modular content to tailor material to individual needs, neurodiverse profiles, and stages of recovery.

### Practical implications

For clinicians, these findings highlight the value of co-produced digital tools as adjunctive supports within care pathways. Within the context of this small, exploratory evaluation, the app appeared to help bridge gaps between appointments, enhance health literacy, support difficult conversations, and encourage early help-seeking, particularly among younger users or those hesitant to approach services. Managing expectations and guiding users on how to engage with the app may be important to reduce the risk of information overload and ensure that digital support complements, rather than replaces, evidence-based treatment. For developers, the results point to opportunities to strengthen usability through greater personalisation, a broader range of media formats, enhanced accessibility features and clearer signposting tailored to different stages of recovery. The consistent value that participants placed on trustworthy information and inclusive, non-judgemental language reinforces the importance of continued clinical oversight, meaningful co-production, and fidelity to recognised quality standards. At a service and commissioning level, evidence-informed digital tools offer a scalable means to support early identification, provide safer alternatives to harmful online content, and extend continuity of support for individuals and families affected by eating disorders. Integrating such tools within stepped-care and blended-care models may help widen access, particularly in contexts of long waiting times and limited specialist provision.

## Strengths and limitations

A key strength of this study is its mixed-methods design, which enabled integration of quantitative indicators of satisfaction and perceived usefulness with in-depth interviews with individuals with lived experience and healthcare professionals. This integration strengthened the interpretation of findings by allowing convergent and divergent perspectives to be explored and located explanatory power in qualitative accounts while using quantitative metrics to contextualise findings. Credibility was further enhanced by the analysis being conducted by a health psychologist who was not involved in the app’s development, reducing the likelihood of developer bias, and by PPIE involvement in reviewing and shaping the final category structure.

However, several limitations should be considered. The sample was small (*n* = 16 survey; *n* = 4 interviews) and predominantly White British/White Other, which constrains the transferability of findings to more diverse populations. All findings should be treated as exploratory rather than conclusive. Participation was voluntary, raising the possibility that individuals with more positive experiences or higher engagement were overrepresented. No minimum engagement threshold was set, meaning variation in depth of engagement likely influenced responses. The exploratory engagement comparison (Table 4) indicated a directional pattern favouring higher-engagement respondents, but group sizes preclude any reliable interpretation beyond hypothesis generation. Survey and interview participants may also differ in characteristics from the wider app user base. The cross-sectional design limits insight into how perceptions and engagement might change over time, and no clinical outcomes were measured. Granular usage data are not collected by the app in keeping with its privacy-preserving design, preventing contextualisation of the sample against broader engagement patterns in the wider user base. Future evaluations with larger and more diverse samples, longitudinal follow-up, objective engagement metrics, and clinical outcome measures are needed to better understand the app’s effectiveness, acceptability and sustained use across different user groups.

### Researcher positionality

The analysis was led by the first author (VR), a health psychologist with experience in digital health and behaviour-change services. This background may have shaped coding decisions, including sensitivity to domains such as accessibility and emotional safety. Transparency was promoted through an audit trail, reflexive journalling, and iterative supervisory review of the emerging category structure with co-authors (CR-W, CT) across seven documented consultancy meetings. This supervisory process produced concrete and documented analytic changes. Following presentation of a preliminary category structure in April 2024, supervisory feedback identified that the initial labels were organised around app features rather than participant experience, reflecting an unexamined assumption on the part of the lead analyst. In response, all four category labels were revised: for example, “App benefits and impact” became “Supporting understanding, confidence and empowerment.” An initial subcategory addressing app interactivity was also consolidated into the broader personalisation subcategory following supervisory identification of conceptual overlap.

These changes were documented in the consultancy meeting records and illustrate how supervisory engagement shaped analytic outputs rather than simply providing procedural oversight, consistent with quality standards for conventional qualitative content analysis, in which rigour is achieved through systematic procedure, documented decision-making and iterative peer review. CT’s supervisory involvement during the analytic phase meant that lived-experience perspectives were present within the category development process itself, providing an ongoing check on whether the emerging structure resonated with lived experience of eating disorder support. This reflects an understanding of co-production as holding epistemic value at every phase of health research [62], and future evaluations should seek to extend this further, for example through co-analysis of qualitative data.

## Conclusion

This evaluation demonstrates that a co-produced, evidence-informed, nationally recommended and quality-assured mHealth app can offer accessible, trustworthy and emotionally supportive resources for people affected by eating disorders and those who support them. Within this small, exploratory sample, participants valued the app’s clarity, inclusivity and alignment with clinical guidance, and reported benefits for confidence, understanding and reassurance. The overarching pattern, consistent across quantitative and qualitative strands, was that the app functioned as a trust-building resource that helped users navigate their support more confidently, a finding that converged across lived-experience and professional perspectives. Areas for further refinement include personalisation, accessibility features and expanded content for diverse experiences. Next steps also include securing sponsorship to ensure the long-term viability of the app and continuing to expand its reach. Further work and ongoing co-production will be crucial in ensuring digital tools are safe, equitable and responsive to the needs of individuals at different stages of their eating disorder journey.

## Supplementary Information

Supplementary Material 1.

## Data Availability

The datasets generated and analysed during this study are not publicly available to preserve anonymity but may be obtained from the corresponding author on reasonable request for verification.

## Abbreviations

ADHD: Attention-deficit/hyperactivity disorder
BMC: BioMed central
BPS: British psychological society
CBT-E: Enhanced cognitive behavioural therapy
CSQ-8: Client satisfaction questionnaire-8
ED: Eating disorder
EDHIT: Eating disorder health integration team
HCP: Healthcare professional
HCPC: Health and care professions council
HRA: Health Research Authority
JED: Journal of eating disorders
MANTRA: Maudsley Model of Anorexia Nervosa Treatment for Adults
mHealth: Mobile health
MARS: Mobile application rating scale
NIHR: National Institute for Health and Care Research
NHS: National health service
PIF: Patient information forum
PPIE: Patient and Public Involvement and Engagement
QCA: Qualitative content analysis
SUS: System usability scale
UWE: University of the West of England
