# Supplementary material for: User experience and perceived impact of the Eating Disorder Support App: a mixed-methods evaluation
Source: J Eat Disord. 2026 Jun 24;14:213. doi: 10.1186/s40337-026-01655-1 (PMC13560362; doi:10.1186/s40337-026-01655-1)
Supplement: Supplementary file 1 [file 40337_2026_1655_MOESM1_ESM.docx]

**1: Survey instrument**

**EDHIT App Evaluation Questionnaire**

**Introduction**

**Welcome to the Eating Disorder Support App Evaluation Survey**

The Eating Disorder Health Integration Team (EDHIT) has created this survey to evaluate how effective and easy to use the Eating Disorder Support App is. Your feedback is crucial for improving the app so that we can provide even better support for individuals impacted by eating disorders.

**Who can take part in this survey?**

We would like anyone who has used the Eating Disorder Support App to share their thoughts through this survey. You do not have to have experience of an eating disorder or disordered eating to participate. It is important to note that your participation is voluntary, and you do not have to take part.

**Why is your input important?** 
Your valuable insights will directly impact the future development of the Eating Disorder Support App so that it better serves the needs of those affected by eating disorders and the healthcare professionals supporting them. We will analyse all survey responses and use this information to make it more user-friendly, informative, and supportive.

**What can you expect?** 
In this survey we will ask you about your experiences and challenges when using the app, as well as any suggestions you have about how to improve the app's content and how it works.

**Looking after yourself** 
We will ask you about your experiences of using the app in this survey, which may be triggering for some individuals. If you feel uncomfortable or distressed, feel free to stop at any point and please consider reaching out for support. You can find a range of places to get support in the ‘Support’ section of the Eating Disorder Support App.

**How long will it take to complete the survey?**

Completing this survey will take approximately 10-15 minutes of your time.

All your responses will remain confidential and anonymous. This means that no one (including EDHIT) will know your name and we won’t tell anyone outside EDHIT what has been shared in our survey. We want you to feel safe and encouraged to be honest you’re your answers.

Note: When we use the term ‘eating disorders’ in this survey, we are referring to diagnosed eating disorders and undiagnosed disordered eating. We recognise that there is unequal access to diagnosis due to bias and discrimination, and not having a diagnosis doesn’t mean that someone is struggling less.

If you have any questions, please feel free to email EDHIT at [helloedhit@gmail.com](mailto:helloedhit@gmail.com)

Thank you for your willingness to share your insights.

**Section 1: App Usage**

**Using the app**

In this section, we're interested in understanding how you've been using the app and what motivated you to start using it.

1. How often do you use the Eating Disorder Support App for information on eating disorders?

- Daily
- Several times a week
- Once a week
- Several times a month
- Rarely
- Never used

2. How long have you been using the Eating Disorder Support App?

- Less than a month
- 1-3 months
- 3-6 months
- 6-12 months
- More than a year

3. What is your primary reason for using the Eating Disorder Support app? (Select all that apply)

- To gain knowledge about eating disorders
- To understand symptoms and effects
- To find out more about treatment
- To access resources
- To provide support to an individual with lived experience
- For self-help
- To find out who is at risk of getting an eating disorder
- Other (please specify): ____________________

**Section 2: Impact assessment**

In this section, we want to understand the impact of the Eating Disorder Support App on well-being and support. Your honest answers will help us to provide meaningful support and guidance. We encourage you to take a moment to revisit the app if you haven't done so recently, as it may help refresh your memory and provide more accurate feedback.

**Perceived effectiveness**

4. In your experience, what are the positive aspects or strengths of the app that you find particularly beneficial or effective? Please provide details or examples. [Open text]

5. How likely do you think this app is to help increase awareness of disordered eating habits?

- Highly likely
- Likely
- Neutral
- Unlikely
- Highly unlikely

**Knowledge**

6. How likely do you think this app is to increase knowledge/understanding of managing and overcoming eating disorders?

- Highly likely
- Likely
- Neutral
- Unlikely
- Highly unlikely

**Attitudes**

7. How likely do you think this app is to change attitudes toward overcoming disordered eating behaviours?

- Highly likely
- Likely
- Neutral
- Unlikely
- Highly unlikely

**Intention to change**

8. How likely do you think this app is to increase intentions/motivation to address and overcome disordered eating habits?

- Highly likely
- Likely
- Neutral
- Unlikely
- Highly unlikely

**Help-seeking**

9. How likely do you think the use of this app is to encourage further help-seeking for individuals dealing with eating disorders (if it’s required)?

- Highly likely
- Likely
- Neutral
- Unlikely
- Highly unlikely

**Behaviour change**

10. How likely do you think the use of this app is to contribute to positive behaviour changes in managing and overcoming disordered eating?

- Highly likely
- Likely
- Neutral
- Unlikely
- Highly unlikely

**Support**

11. How likely do you think the use of this app is to contribute to feeling more equipped or knowledgeable about supporting a loved one/patients dealing with an eating disorder?

- Highly likely
- Likely
- Neutral
- Unlikely
- Highly unlikely

12. If applicable, how has using the app affected you personally?

**Concerns**

13. Do you have any concerns about the information provided by the app?

- Highly concerned
- Concerned
- Neutral
- Not very concerned
- Not concerned at all

- If applicable, what are your concerns with the app? [Open text]

**Section 3: App experience**

In this section, we'd like to delve into your experiences with using the app. Please provide your honest opinions and experiences. We encourage you to take a moment to revisit the app if you haven'tdone so recently, as it may help refresh your memory and provide more accurate feedback.

**Engagement**

14. How useful is the information about eating disorders in the app?

- Not useful at all
- Slightly useful
- Moderately useful
- Very useful
- Extremely useful

15. How useful are the tools for supporting yourself or someone else with an eating disorder in the app?

- Not useful at all
- Slightly useful
- Moderately useful
- Very useful
- Extremely useful

- Which area(s) of the app do you find most useful (tick as many as you like)?

16. Local page (if listed)
17. About EDs
18. Treatment
19. Support
20. Partners
21. Self-help
22. At Risk
23. Calm Zone
24. Supporting others
25. Other (please specify): ____________________

- What do you like about the sections you've ticked? [Open text]

26. How interesting and engaging is the app in capturing and maintaining your interest?

- Not interesting at all
- Slightly interesting
- Moderately interesting
- Very interesting
- Extremely interesting

**Functionality**

27. Overall, how would you rate the functionality of the app (i.e. how well it does what it’s supposed to)?

- Very poor
- Poor
- Average
- Good
- Excellent

28. How intuitive is the app's navigation and screen changes?

- Very confusing
- Somewhat confusing
- Neutral
- Mostly intuitive
- Extremely intuitive

**Aesthetics**

29. How visually appealing do you find the app?

- Not appealing at all
- Slightly appealing
- Moderately appealing
- Very appealing
- Extremely appealing

**Information Quality**

30. To the best of your knowledge, how accurate and reliable is the information presented in the app?

- Very inaccurate/unreliable
- Inaccurate/unreliable
- Neutral
- Accurate/reliable
- Very accurate/reliable

31. How relevant is the information in the app to eating disorders?

- Not relevant at all
- Slightly relevant
- Neutral
- Mostly relevant
- Highly relevant

32. How would you rate the understandability of the language used in the app?

- Not relevant at all
- Slightly relevant
- Neutral
- Mostly relevant
- Highly relevant

33. How would you rate the understandability of the language used in the app?

- Very clear and easy to understand
- Clear and mostly understandable
- Neither clear nor unclear
- Somewhat unclear and challenging to understand
- Very unclear and difficult to understand

34. How would you describe the tone of voice used in the app? [open text]

35. How would you rate the comprehensiveness of the provided information?

- Not comprehensive at all
- Slightly comprehensive
- Moderately comprehensive
- Very comprehensive
- Extremely comprehensive

36. How sensitive is the information presented to the needs and concerns of individuals with eating disorders/disordered eating?

- Not sensitive at all
- Slightly sensitive
- Moderately sensitive
- Very sensitive
- Extremely sensitive

37. To what extent do you feel the app includes a diverse range of perspectives and experiences about eating disorders?

- Not at all inclusive of diverse perspectives and experiences
- Slightly inclusive of diverse perspectives and experiences
- Moderately inclusive of diverse perspectives and experiences
- Very inclusive of diverse perspectives and experiences
- Extremely inclusive of diverse perspectives and experiences

38. Are there any aspects of the app that you think could be more inclusive?

**Section 5: Overall satisfaction & improvements**

This section focuses on your overall satisfaction with the Eating Disorder Support App and ideas for improvements. Your honest feedback will guide us in enhancing the app's features and services to better meet your needs. Thank you for taking the time to share your thoughts.

39. How would you rate the quality of the app?

- Excellent
- Good
- Fair
- Poor
- Very Poor

40. Did the app offer the type of support or information you were looking for? (reverse order)

- Yes, definitely
- Yes, generally
- I’m not sure
- Not particularly
- Not at all

41. To what extent has the app met your needs?

- Almost all of my needs have been met
- Most of my needs have been met
- Only a few of my needs have been met
- None of my needs have been met
- Not applicable

42. If a friend were dealing with eating disorders, would you recommend this app to them? (reverse order)

- Yes, definitely
- Yes, I think so
- I’m not sure
- Not particularly
- Not at all

43. Has the app helped you to deal more effectively with eating disorder-related challenges (if applicable)?

- Yes, it helped a great deal
- Yes, it helped somewhat
- No, it really didn’t help
- No, it seemed to make things worse
- Not applicable

44. If you were to seek help again, would you choose to use this app? (reverse order)

- Yes, definitely
- Yes, I think so
- I’m not sure
- Not particularly
- Not at all

45. What specific improvements or features would you suggest for the Eating Disorder Support App? [Open text]

46. Are there any specific topics or information you wish were included or improved within the app? [Open text]

47. Do you have any additional comments, suggestions, or feedback regarding the Eating Disorder Support App that you would like to share with us? [Open text]

**Section 6: Demographics**

In order to better understand our diverse user community, we'd appreciate if you could share a bit about yourself:

1. How old are you?

48. Under 25
49. 25-34
50. 35-44
51. 45-54
52. 55-64
53. 65 and above

2. How would you describe your gender?

- Male
- Female
- Non-binary
- Prefer not to say
- Other (please specify)

3. How would you describe your ethnicity?

- Asian/Asian British (please specify) [Open text]
- Black/African/Caribbean/Black British (please specify) [Open text]
- Mixed/Multiple ethnic groups (please specify) [Open text]
- Other ethnic group (please specify) [Open text]
- White British
- White Other (please specify) [Open text]
- Prefer not to say

4. What area do you live in?

- Bath
- Bristol
- Northeast Somerset
- North Somerset
- South Gloucestershire
- Other (please specify)

5. Are you a health professional?

- Yes
- No

If yes, please specify your occupation (for health professionals): [Open text]

5. If you feel comfortable sharing, how would you describe your journey in relation to eating disorders?

- I have a personal experience with an eating disorder.
- I have supported a loved one with an eating disorder.
- I have both personal and supportive experiences.
- I have no direct experience but seek information for understanding.
- Prefer not to say.

**Section 7: Next steps**

Would you be interested in participating in a follow-up interview or focus group to discuss your experiences with the app in more detail?

    - Yes, I would be interested

    - No, I'm not interested

Are there any specific aspects or topics you would like to discuss in-depth during an interview or focus group? [Open text]

If yes, please provide your contact information (preferred name, email/phone number) for further communication, a member of EDHIT will be in touch with you in due course (this information will be held strictly confidential by EDHIT and will be deleted once the evaluation is complete).  [Open text]

**Debrief and thank you**

**2: Interview topic guide**

**Objective:** The objective of the follow-up interview is to gather more in-depth insights and feedback from respondents who have completed the Eating Disorder Support App Evaluation Survey.

**Duration:** Approximately 30-45 minutes

**Location:** MS Teams

**Semi-structured interview schedule for individuals with lived experience:**

**Agenda:**

**Note:** *The interview schedule may be adjusted based on the flow of the conversation and specific areas of interest or concern expressed by the respondent. Flexibility is key to ensuring a productive and engaging discussion.*

**Introduction (5 minutes)**

- Greet the respondent and thank them for their willingness to participate in the follow-up interview.
- Introduce yourself and explain who you are in relation to the ED support app evaluation.
- Briefly explain the purpose of the interview: to gather more in-depth feedback on their experiences with the Eating Disorder Support App.
- Reiterate the confidentiality and voluntary nature of participation.
- If you decide you would like to withdraw from this interview part of the evaluation after we’ve finished the interview, you can do so by emailing us up to 4 weeks after the date of your interview. After this we won’t be able to remove your interview from our evaluation because it will have been anonymised and so won’t be identifiable.
- Emphasise to the respondent that we (the evaluation team) want this interview to be a positive experience for you, so if there are any questions that make you don’t feel comfortable to answer please just say and we can easily move on to another one.
- Emphasise to the respondent that you’re interested in their honest feedback, regardless of whether this is negative, positive, or neutral.
- Check if they’re still happy for the interview to be recorded and if they have any questions.

**Background information (5mins)**

- What motivated you to use the ED Support app?
- Can you tell me about your experience with the eating disorder support app?

**Initial impressions (5mins)**

- How would you describe the app?
- What were your initial thoughts when you first downloaded the app?
- Did the app meet your expectations? Why or why not?
- Were there any specific features or benefits that stood out to you?
- How useful were the information and resources on the app relating to eating disorders?

**Impact (15 mins)**

- How has the app impacted you personally since you began using it?
- Can you discuss any changes you've experienced as a result of using the app?
- Are there any specific instances where the app has made a difference in your journey with an eating disorder?
- In what ways has the app affected your emotional well-being and mental health?
- Can you tell me about any changes that you’ve noticed in your behaviour since using the app?
- Can you tell me about any changes that you’ve noticed in your mindset since using the app?
- How has the app impacted you in terms of coping strategies for managing the eating disorder?
- Can you provide examples of how you've applied these strategies in your daily life?

If needed, probe further with the questions from the survey **Impact assessment below.**

- Can you provide examples of how the app has made you more aware of disordered eating behaviours?
- How has the app helped you understand eating disorders?
- In your opinion, how has the app influenced how you feel about disordered eating behaviours?
- Do you believe the app can help address and overcome disordered eating habits? If so, why?
- How likely do you think the app encourages individuals to seek help for eating disorders?

**Accessibility, usability**

- Can you tell me about how easy or difficult you found it to understand the language used in the app?
- How easy or difficult was it to find your way around the app?
- How satisfied/unsatisfied are you with the usability of the app? Can you tell me more about why you are satisfied/unsatisfied?
- Can you talk to me about how socially and culturally inclusive or not inclusive you think the app is?

**Recommendations and future use (5 minutes)**

- Would you recommend this app to others struggling with eating disorders? Why or why not?
- Do you plan to continue using the app in the future? Why or why not?
- Do you have any suggestions for improving the app or adding new features?
- Is there anything else you would like to share about your experience with the app, or that you feel would make the app more useful?

If time allows additional questions:

Usability and user interface

- How intuitive did you find the app menus and features to use?
- What challenges, if any, did you encounter while using the app?
- Which features of the app did you find most useful?
- Were there any features that you felt were lacking or could be improved?

**Closing**

- Thank the respondent for their time and input.
- Remind them of the confidentiality of the interview data.
- Provide contact information for follow-up questions or additional feedback.

**Semi-structured interview schedule for health professionals:**

**Agenda:**

**Note:** *The interview schedule may be adjusted based on the flow of the conversation and specific areas of interest or concern expressed by the respondent. Flexibility is key to ensuring a productive and engaging discussion.*

**Introduction (5 minutes):**

- Welcome the respondent and express appreciation for their expertise and insights.
- Introduce yourself and explain who you are in relation to the ED support app evaluation.
- Explain that the purpose of the interview is to gather their professional perspective on the effectiveness and impact of the Eating Disorder Support App.
- Reiterate the confidentiality and voluntary nature of participation. Emphasise to the respondent that we (the evaluation team) want this interview to be a positive experience for you, so if there are any questions that make you don’t feel comfortable to answer please just say and we can easily move on to another one.
- Emphasise to the respondent that you’re interested in their honest feedback, regardless of whether this is negative, positive, or neutral.
- If you decide you would like to withdraw from this interview part of the evaluation after we’ve finished the interview, you can do so by emailing us up to 4 weeks after the date of your interview. After this we won’t be able to remove your interview from our evaluation because it will have been anonymised and so won’t be identifiable.
- Check if they’re still happy for the interview to be recorded and if they have any questions.

**Background information (5 minutes)**

- Can you tell me about your experience with the eating disorder support app?
- What motivated you to use the ED Support app in your work, or recommend it to people?

**Initial impressions (5 minutes)**

- How would you describe the app?
- What were your initial thoughts or impressions when you first downloaded the app?
- Did the app meet your expectations? Why or why not?
- Were there any specific features that stood out to you?
- How did you find the information and resources related to eating disorders?

**Impact (15 minutes)**

- How has the app impacted your professional practice since you began using it?
- Can you discuss any changes you've experienced as a result of using the app?
  - How has the app impacted your professional knowledge and understanding of managing eating disorders?
  - In what ways has the app influenced your approach to treating patients with eating disorders?
  - How has the app provided you with any tools or strategies for managing and addressing eating disorders in your patients?
  - Have you noticed any changes in patient engagement or outcomes since incorporating the app into your practice?
  - Can you share any specific instances where the app has played a role in supporting your patients' journeys?
- If yes prompts below:
- In what ways has the app affected their emotional well-being and mental health?
- Can you tell me about any changes that you’ve noticed in their behaviour since using the app?
- Can you tell me about any changes that you’ve noticed in their mindset since using the app?
- How has the app impacted them in terms of coping strategies for managing the eating disorder?
- Can you provide examples of how they've applied these strategies in their daily life?

**Accessibility, usability**

- Can you tell me about how easy you found it to understand the language used in the app?
- How easy or difficult was it to find your way around the app?
- How satisfied are you with the usability of the app? Can you tell me more about why you are satisfied/unsatisfied?
- Can you talk to me about how socially and culturally inclusive you think the app is?

**Recommendations and future use (5 minutes)**

- Would you recommend this app to patients struggling with eating disorders? Why or why not?
- Based on your professional experience, would you recommend this app to other healthcare professionals working with patients with eating disorders?
- Do you plan to continue using the app in your practice in the future? Why or why not?
- Do you have any suggestions for improving the app or adding new features to better support patients?
- Do you have any suggestions for improving the app or adding new features to better support health professionals?
- Is there anything else you would like to share about your experience with the app?

If time allows additional questions:

Usability and user interface

- How intuitive did you find the app menus and features to use?
- What challenges, if any, did you encounter while using the app?
- Which features of the app did you find most useful?
- Were there any features that you felt were lacking or could be improved?

**Closing**

- Thank the respondent for their time and valuable insights.
- Reiterate the confidentiality of the interview data.
- Provide contact information for any follow-up questions or additional feedback.

**Semi-structured interview schedule for individuals supporting someone with lived experience**

**Agenda:**

**Note:** *The interview schedule may be adjusted based on the flow of the conversation and specific areas of interest or concern expressed by the respondent. Flexibility is key to ensuring a productive and engaging discussion.*

**Introduction (5 minutes):**

- Welcome the respondent and express appreciation for their expertise and insights.
- Introduce yourself and explain who you are in relation to the ED support app evaluation.
- Explain that the purpose of the interview is to gather their professional perspective on the effectiveness and impact of the Eating Disorder Support App.
- Reiterate the confidentiality and voluntary nature of participation. Emphasise to the respondent that we (the evaluation team) want this interview to be a positive experience for you, so if there are any questions that make you don’t feel comfortable to answer please just say, and we can easily move on to another one.
- Emphasise to the respondent that you’re interested in their honest feedback, regardless of whether this is negative, positive, or neutral.
- If you decide you would like to withdraw from this interview part of the evaluation after we’ve finished the interview, you can do so by emailing us up to 4 weeks after the date of your interview. After this we won’t be able to remove your interview from our evaluation because it will have been anonymised and so won’t be identifiable.
- Check if they’re still happy for the interview to be recorded and if they have any questions.

**Background information (5 minutes)**

- Can you tell me about your experience with the eating disorder support app?
- What motivated you to use the ED Support app?

**Initial impressions (5 minutes)**

- What were your initial thoughts or impressions when you first downloaded the app?
- Did the app meet your expectations? Why or why not?
- Were there any specific features that stood out to you?
- How would you describe the app?
- How did you find the information and resources related to eating disorders?

**Impact (15 minutes)**

- How has the app impacted you since you began using it?
- Can you discuss any changes you've experienced as a result of using the app?
  - How has the app impacted your knowledge and understanding of managing eating disorders?
  - In what ways has the app influenced your approach to supporting others with eating disorders?
  - How has the app provided you with any tools or strategies supporting others with eating disorders?
  - Have you noticed any impact on others with eating disorders since incorporating the app?
  - Can you share any specific instances where the app has played a role in supporting someone with lived experience?
- If yes prompts below:
- In what ways has the app affected their emotional well-being and mental health?
- Can you tell me about any changes that you’ve noticed in their behaviour since using the app?
- Can you tell me about any changes that you’ve noticed in their mindset since using the app?
- How has the app impacted them in terms of coping strategies for managing the eating disorder?
- Can you provide examples of how they've applied these strategies in their daily life?

**Accessibility, usability**

- Can you tell me about how easy you found it to understand the language used in the app?
- How easy or difficult was it to find your way around the app?
- How satisfied are you with the usability of the app? Can you tell me more about why you are satisfied/unsatisfied?
- Can you talk to me about how socially and culturally inclusive you think the app is?

**Recommendations and future use (5 minutes)**

- Would you recommend this app to individuals struggling with eating disorders? Why or why not?
- Based on your experience, would you recommend this app to others supporting individuals with eating disorders? Why or why not?
- Do you plan to continue using the app in your practice in the future? Why or why not?
- Do you have any suggestions for improving the app or adding new features to better support patients?
- Do you have any suggestions for improving the app or adding new features to better support health professionals?
- Is there anything else you would like to share about your experience with the app?

If time allows additional questions:

Usability and user interface

- How intuitive did you find the app menus and features to use?
- What challenges, if any, did you encounter while using the app?
- Which features of the app did you find most useful?
- Were there any features that you felt were lacking or could be improved?

**Closing**

- Thank the respondent for their time and valuable insights.
- Reiterate the confidentiality of the interview data.
- Provide contact information for any follow-up questions or additional feedback.

**3: Supplementary Tables**

**Table 1**

| **Age** | | | | | |
| --- | --- | --- | --- | --- | --- |
|  | | Frequency | Percent | Valid Percent | Cumulative Percent |
| Valid | Under 25 | 2 | 12.5 | 12.5 | 12.5 |
|  | 25-34 | 2 | 12.5 | 12.5 | 25.0 |
|  | 35-44 | 6 | 37.5 | 37.5 | 62.5 |
|  | 45-54 | 4 | 25.0 | 25.0 | 87.5 |
|  | 55-64 | 2 | 12.5 | 12.5 | 100.0 |
|  | Total | 16 | 100.0 | 100.0 |  |

| **Gender** | | | | | |
| --- | --- | --- | --- | --- | --- |
|  | | Frequency | Percent | Valid Percent | Cumulative Percent |
| Valid | Female | 15 | 93.8 | 93.8 | 93.8 |
|  | Prefer not to say | 1 | 6.3 | 6.3 | 100.0 |
|  | Total | 16 | 100.0 | 100.0 |  |

| **Ethnicity** | | | | | |
| --- | --- | --- | --- | --- | --- |
|  | | Frequency | Percent | Valid Percent | Cumulative Percent |
| Valid | White British | 10 | 62.5 | 62.5 | 62.5 |
|  | White Other | 6 | 37.5 | 37.5 | 100.0 |
|  | Total | 16 | 100.0 | 100.0 |  |

| **Area** | | | | | |
| --- | --- | --- | --- | --- | --- |
|  | | Frequency | Percent | Valid Percent | Cumulative Percent |
| Valid | Bath and North East Somerset (BANES) | 2 | 12.5 | 12.5 | 12.5 |
|  | Bristol | 3 | 18.8 | 18.8 | 31.3 |
|  | South Gloucestershire | 2 | 12.5 | 12.5 | 43.8 |
|  | Other | 9 | 56.3 | 56.3 | 100.0 |
|  | Total | 16 | 100.0 | 100.0 |  |

| **Health_professional** | | | | | |
| --- | --- | --- | --- | --- | --- |
|  | | Frequency | Percent | Valid Percent | Cumulative Percent |
| Valid | No | 6 | 37.5 | 37.5 | 37.5 |
|  | Yes | 10 | 62.5 | 62.5 | 100.0 |
|  | Total | 16 | 100.0 | 100.0 |  |

| **ED_experience** | | | | | |
| --- | --- | --- | --- | --- | --- |
|  | | Frequency | Percent | Valid Percent | Cumulative Percent |
| Valid | I have a personal experience with an eating disorder. | 7 | 43.8 | 43.8 | 43.8 |
|  | I have supported someone with an eating disorder. | 4 | 25.0 | 25.0 | 68.8 |
|  | I have both personal and supportive experiences. | 5 | 31.3 | 31.3 | 100.0 |
|  | Total | 16 | 100.0 | 100.0 |  |

**Table 3**

| **Usage_frequency** | | | | | |
| --- | --- | --- | --- | --- | --- |
|  | | Frequency | Percent | Valid Percent | Cumulative Percent |
| Valid | Daily | 1 | 6.3 | 6.3 | 6.3 |
|  | Several times a week | 2 | 12.5 | 12.5 | 18.8 |
|  | Once a week | 3 | 18.8 | 18.8 | 37.5 |
|  | Several times a month | 4 | 25.0 | 25.0 | 62.5 |
|  | Rarely | 6 | 37.5 | 37.5 | 100.0 |
|  | Total | 16 | 100.0 | 100.0 |  |

| **Usage_length** | | | | | |
| --- | --- | --- | --- | --- | --- |
|  | | Frequency | Percent | Valid Percent | Cumulative Percent |
| Valid | Less than a month | 8 | 50.0 | 50.0 | 50.0 |
|  | 1-3 months | 2 | 12.5 | 12.5 | 62.5 |
|  | 3-6 months | 1 | 6.3 | 6.3 | 68.8 |
|  | 6-12 months | 1 | 6.3 | 6.3 | 75.0 |
|  | More than a year | 4 | 25.0 | 25.0 | 100.0 |
|  | Total | 16 | 100.0 | 100.0 |  |

| **UsageReasons Frequencies** | | | | |
| --- | --- | --- | --- | --- |
|  | | Responses | | Percent of Cases |
|  |  | N | Percent |  |
| Reasong for using the app^a^ | Usage_reason_info | 4 | 11.8% | 25.0% |
|  | Usage_reason_symptoms | 3 | 8.8% | 18.8% |
|  | Usage_reason_treatment | 3 | 8.8% | 18.8% |
|  | Usage_reason_resources | 6 | 17.6% | 37.5% |
|  | Usage_reason_support | 6 | 17.6% | 37.5% |
|  | Usage_reason_selfhelp | 5 | 14.7% | 31.3% |
|  | Usage_reason_risk | 1 | 2.9% | 6.3% |
|  | Usage_reason_other | 6 | 17.6% | 37.5% |
| Total | | 34 | 100.0% | 212.5% |
| a. Dichotomy group tabulated at value 1. | | | | |

**Table 5**

| **Quality** | | | | | |
| --- | --- | --- | --- | --- | --- |
|  | | Frequency | Percent | Valid Percent | Cumulative Percent |
| Valid | Excellent | 8 | 50.0 | 50.0 | 50.0 |
|  | Good | 7 | 43.8 | 43.8 | 93.8 |
|  | Fair | 1 | 6.3 | 6.3 | 100.0 |
|  | Total | 16 | 100.0 | 100.0 |  |

| **Support_relevance** | | | | | |
| --- | --- | --- | --- | --- | --- |
|  | | Frequency | Percent | Valid Percent | Cumulative Percent |
| Valid | Not at all | 1 | 6.3 | 6.3 | 6.3 |
|  | Not particularly | 1 | 6.3 | 6.3 | 12.5 |
|  | I'm not sure | 2 | 12.5 | 12.5 | 25.0 |
|  | Yes, generally | 7 | 43.8 | 43.8 | 68.8 |
|  | Yes, definitely | 5 | 31.3 | 31.3 | 100.0 |
|  | Total | 16 | 100.0 | 100.0 |  |

| **Needs** | | | | | |
| --- | --- | --- | --- | --- | --- |
|  | | Frequency | Percent | Valid Percent | Cumulative Percent |
| Valid |  | 3 | 18.8 | 18.8 | 18.8 |
|  | Almost all of my needs have been met | 5 | 31.3 | 31.3 | 50.0 |
|  | Most of my needs have been met | 3 | 18.8 | 18.8 | 68.8 |
|  | Only a few of my needs have been met | 3 | 18.8 | 18.8 | 87.5 |
|  | None of my needs have been met | 2 | 12.5 | 12.5 | 100.0 |
|  | Total | 16 | 100.0 | 100.0 |  |

| **Recomendation** | | | | | |
| --- | --- | --- | --- | --- | --- |
|  | | Frequency | Percent | Valid Percent | Cumulative Percent |
| Valid | Not particularly | 1 | 6.3 | 6.3 | 6.3 |
|  | I'm not sure | 1 | 6.3 | 6.3 | 12.5 |
|  | Yes, I think so | 5 | 31.3 | 31.3 | 43.8 |
|  | Yes, definitely | 9 | 56.3 | 56.3 | 100.0 |
|  | Total | 16 | 100.0 | 100.0 |  |

| **Reusability** | | | | | |
| --- | --- | --- | --- | --- | --- |
|  | | Frequency | Percent | Valid Percent | Cumulative Percent |
| Valid | Not particularly | 2 | 12.5 | 12.5 | 12.5 |
|  | I'm not sure | 2 | 12.5 | 12.5 | 25.0 |
|  | Yes, I think so | 7 | 43.8 | 43.8 | 68.8 |
|  | Yes, definitely | 5 | 31.3 | 31.3 | 100.0 |
|  | Total | 16 | 100.0 | 100.0 |  |

**Table 6**

| **Information** | | | | | |
| --- | --- | --- | --- | --- | --- |
|  | | Frequency | Percent | Valid Percent | Cumulative Percent |
| Valid |  | 1 | 6.3 | 6.3 | 6.3 |
|  | Moderately useful | 5 | 31.3 | 31.3 | 37.5 |
|  | Very useful | 4 | 25.0 | 25.0 | 62.5 |
|  | Extremely useful | 6 | 37.5 | 37.5 | 100.0 |
|  | Total | 16 | 100.0 | 100.0 |  |

| **Accuracy** | | | | | |
| --- | --- | --- | --- | --- | --- |
|  | | Frequency | Percent | Valid Percent | Cumulative Percent |
| Valid | Very inaccurate/unreliable | 1 | 6.3 | 6.3 | 6.3 |
|  | I don't know | 3 | 18.8 | 18.8 | 25.0 |
|  | Accurate/reliable | 6 | 37.5 | 37.5 | 62.5 |
|  | Very accurate/reliable | 6 | 37.5 | 37.5 | 100.0 |
|  | Total | 16 | 100.0 | 100.0 |  |

| **Relevance_information** | | | | | |
| --- | --- | --- | --- | --- | --- |
|  | | Frequency | Percent | Valid Percent | Cumulative Percent |
| Valid | Neutral | 1 | 6.3 | 6.3 | 6.3 |
|  | Mostly relevant | 5 | 31.3 | 31.3 | 37.5 |
|  | Highly relevant | 10 | 62.5 | 62.5 | 100.0 |
|  | Total | 16 | 100.0 | 100.0 |  |

| **Comprehensiveness** | | | | | |
| --- | --- | --- | --- | --- | --- |
|  | | Frequency | Percent | Valid Percent | Cumulative Percent |
| Valid | Moderately comprehensive | 6 | 37.5 | 37.5 | 37.5 |
|  | Very comprehensive | 5 | 31.3 | 31.3 | 68.8 |
|  | Extremely comprehensive | 5 | 31.3 | 31.3 | 100.0 |
|  | Total | 16 | 100.0 | 100.0 |  |

**Table 7**

| **Awareness** | | | | | |
| --- | --- | --- | --- | --- | --- |
|  | | Frequency | Percent | Valid Percent | Cumulative Percent |
| Valid | Very impactful | 4 | 25.0 | 25.0 | 25.0 |
|  | Moderately impactful | 6 | 37.5 | 37.5 | 62.5 |
|  | Neutral | 4 | 25.0 | 25.0 | 87.5 |
|  | Not at all impactful | 2 | 12.5 | 12.5 | 100.0 |
|  | Total | 16 | 100.0 | 100.0 |  |

| **Knowledge** | | | | | |
| --- | --- | --- | --- | --- | --- |
|  | | Frequency | Percent | Valid Percent | Cumulative Percent |
| Valid | Very effective | 4 | 25.0 | 25.0 | 25.0 |
|  | Moderately effective | 5 | 31.3 | 31.3 | 56.3 |
|  | Neutral | 5 | 31.3 | 31.3 | 87.5 |
|  | Not effective | 2 | 12.5 | 12.5 | 100.0 |
|  | Total | 16 | 100.0 | 100.0 |  |

| **Intention** | | | | | |
| --- | --- | --- | --- | --- | --- |
|  | | Frequency | Percent | Valid Percent | Cumulative Percent |
| Valid | Highly likely | 3 | 18.8 | 18.8 | 18.8 |
|  | Likely | 10 | 62.5 | 62.5 | 81.3 |
|  | Neutral | 1 | 6.3 | 6.3 | 87.5 |
|  | Unlikely | 2 | 12.5 | 12.5 | 100.0 |
|  | Total | 16 | 100.0 | 100.0 |  |

| **Help_seeking** | | | | | |
| --- | --- | --- | --- | --- | --- |
|  | | Frequency | Percent | Valid Percent | Cumulative Percent |
| Valid | Highly likely | 5 | 31.3 | 31.3 | 31.3 |
|  | Likely | 9 | 56.3 | 56.3 | 87.5 |
|  | Neutral | 2 | 12.5 | 12.5 | 100.0 |
|  | Total | 16 | 100.0 | 100.0 |  |

| **Behaviour_change** | | | | | |
| --- | --- | --- | --- | --- | --- |
|  | | Frequency | Percent | Valid Percent | Cumulative Percent |
| Valid | Highly likely | 1 | 6.3 | 6.3 | 6.3 |
|  | Likely | 10 | 62.5 | 62.5 | 68.8 |
|  | Neutral | 4 | 25.0 | 25.0 | 93.8 |
|  | Unlikely | 1 | 6.3 | 6.3 | 100.0 |
|  | Total | 16 | 100.0 | 100.0 |  |

**Table 8**

| **Effectiveness_supporting** | | | | | |
| --- | --- | --- | --- | --- | --- |
|  | | Frequency | Percent | Valid Percent | Cumulative Percent |
| Valid | Highly likely | 8 | 50.0 | 50.0 | 50.0 |
|  | Likely | 5 | 31.3 | 31.3 | 81.3 |
|  | Neutral | 2 | 12.5 | 12.5 | 93.8 |
|  | Unlikely | 1 | 6.3 | 6.3 | 100.0 |
|  | Total | 16 | 100.0 | 100.0 |  |

| **Help** | | | | | |
| --- | --- | --- | --- | --- | --- |
|  | | Frequency | Percent | Valid Percent | Cumulative Percent |
| Valid |  | 7 | 43.8 | 43.8 | 43.8 |
|  | Yes, it helped a great deal | 5 | 31.3 | 31.3 | 75.0 |
|  | Yes, it helped somewhat | 2 | 12.5 | 12.5 | 87.5 |
|  | No, it really didn’t help | 2 | 12.5 | 12.5 | 100.0 |
|  | Total | 16 | 100.0 | 100.0 |  |

**Table 9**

| **Navigation** | | | | | |
| --- | --- | --- | --- | --- | --- |
|  | | Frequency | Percent | Valid Percent | Cumulative Percent |
| Valid | Somewhat confusing | 1 | 6.3 | 6.3 | 6.3 |
|  | Neutral | 1 | 6.3 | 6.3 | 12.5 |
|  | Mostly intuitive | 10 | 62.5 | 62.5 | 75.0 |
|  | Extremely intuitive | 4 | 25.0 | 25.0 | 100.0 |
|  | Total | 16 | 100.0 | 100.0 |  |

| **Appeal** | | | | | |
| --- | --- | --- | --- | --- | --- |
|  | | Frequency | Percent | Valid Percent | Cumulative Percent |
| Valid | Not appealing at all | 1 | 6.3 | 6.3 | 6.3 |
|  | Slightly appealing | 2 | 12.5 | 12.5 | 18.8 |
|  | Moderately appealing | 3 | 18.8 | 18.8 | 37.5 |
|  | Very appealing | 5 | 31.3 | 31.3 | 68.8 |
|  | Extremely appealing | 5 | 31.3 | 31.3 | 100.0 |
|  | Total | 16 | 100.0 | 100.0 |  |

**Table 10**

| **Engagement** | | | | | |
| --- | --- | --- | --- | --- | --- |
|  | | Frequency | Percent | Valid Percent | Cumulative Percent |
| Valid | Not interesting at all | 3 | 18.8 | 18.8 | 18.8 |
|  | Slightly interesting | 1 | 6.3 | 6.3 | 25.0 |
|  | Moderately interesting | 4 | 25.0 | 25.0 | 50.0 |
|  | Very interesting | 6 | 37.5 | 37.5 | 87.5 |
|  | Extremely interesting | 2 | 12.5 | 12.5 | 100.0 |
|  | Total | 16 | 100.0 | 100.0 |  |

| **Functionality** | | | | | |
| --- | --- | --- | --- | --- | --- |
|  | | Frequency | Percent | Valid Percent | Cumulative Percent |
| Valid | Poor | 1 | 6.3 | 6.3 | 6.3 |
|  | Average | 2 | 12.5 | 12.5 | 18.8 |
|  | Good | 8 | 50.0 | 50.0 | 68.8 |
|  | Excellent | 5 | 31.3 | 31.3 | 100.0 |
|  | Total | 16 | 100.0 | 100.0 |  |

| **$AppFeatures Frequencies** | | | | |
| --- | --- | --- | --- | --- |
|  | | Responses | | Percent of Cases |
|  |  | N | Percent |  |
| Most useful parts of the app^a^ | Local_page | 3 | 4.3% | 20.0% |
|  | About_ED | 7 | 10.0% | 46.7% |
|  | At_risk | 5 | 7.1% | 33.3% |
|  | Life | 6 | 8.6% | 40.0% |
|  | Self_help | 12 | 17.1% | 80.0% |
|  | Treatment | 6 | 8.6% | 40.0% |
|  | Support | 10 | 14.3% | 66.7% |
|  | Calm_zone | 9 | 12.9% | 60.0% |
|  | Supporting_others | 8 | 11.4% | 53.3% |
|  | Partners | 3 | 4.3% | 20.0% |
|  | Other | 1 | 1.4% | 6.7% |
| Total | | 70 | 100.0% | 466.7% |
| a. Dichotomy group tabulated at value 1. | | | | |

**Table 11**

| **Concerns** | | | | | |
| --- | --- | --- | --- | --- | --- |
|  | | Frequency | Percent | Valid Percent | Cumulative Percent |
| Valid | Neutral | 3 | 18.8 | 18.8 | 18.8 |
|  | Not very concerned | 2 | 12.5 | 12.5 | 31.3 |
|  | Not concerned at all | 11 | 68.8 | 68.8 | 100.0 |
|  | Total | 16 | 100.0 | 100.0 |  |

**Table 12**

| **Sensitive** | | | | | |
| --- | --- | --- | --- | --- | --- |
|  | | Frequency | Percent | Valid Percent | Cumulative Percent |
| Valid | Moderately sensitive | 2 | 12.5 | 12.5 | 12.5 |
|  | Very sensitive | 8 | 50.0 | 50.0 | 62.5 |
|  | Extremely sensitive | 6 | 37.5 | 37.5 | 100.0 |
|  | Total | 16 | 100.0 | 100.0 |  |

| **Diversity** | | | | | |
| --- | --- | --- | --- | --- | --- |
|  | | Frequency | Percent | Valid Percent | Cumulative Percent |
| Valid | Slightly inclusive of diverse perspectives and experiences | 2 | 12.5 | 12.5 | 12.5 |
|  | Moderately inclusive of diverse perspectives and experiences | 6 | 37.5 | 37.5 | 50.0 |
|  | Very inclusive of diverse perspectives and experiences | 5 | 31.3 | 31.3 | 81.3 |
|  | Extremely inclusive of diverse perspectives and experiences | 3 | 18.8 | 18.8 | 100.0 |
|  | Total | 16 | 100.0 | 100.0 |  |

**Table 13**

| **Language** | | | | | |
| --- | --- | --- | --- | --- | --- |
|  | | Frequency | Percent | Valid Percent | Cumulative Percent |
| Valid | Very clear and easy to understand | 11 | 68.8 | 68.8 | 68.8 |
|  | Clear and mostly understandable | 4 | 25.0 | 25.0 | 93.8 |
|  | Neither clear nor unclear | 1 | 6.3 | 6.3 | 100.0 |
|  | Total | 16 | 100.0 | 100.0 |  |

**4: Analytic documentation**

**Supplementary Table S1. Themes, Subthemes, Definitions and Illustrative Quotes**

| **Theme** | **Subtheme** | **Definition / boundary** | **Illustrative verbatim quotes** |
| --- | --- | --- | --- |
| 1. Supporting understanding, confidence and empowerment | 1.1 Easy access, satisfaction and integration with care | Describes users’ perceptions of the app as easy to navigate, accessible at any time, and a helpful adjunct to clinical support.  Does NOT include reflections on specific content, self-help features, or confidence-building (covered in later subthemes). | “I enjoy what it does… having that information readily available is really important.” (P1)  “You can access it whenever you need it… I’d really push for people to engage with the app.” (P3)  “It crosses over quite nicely with our treatment pathways.” (P1) “Sometimes your appointment might be pushed back… I find it very helpful.” (P3)  “I'd recommend it to other health professionals and to clients or family members.” (P2) “Innovative… providing carers or loved ones with information and easy sign-posting.” (Survey) |
|  | 1.2 Trustworthy information offering something ‘extra’ | Reflects perceptions that the app provides accurate, comprehensive, and reliable information that reduces the need for potentially harmful online searching. Also captures the sense that the app offers an “extra” resource complementing clinical care. Does NOT include emotional reassurance (covered in 1.4) or behaviour change (1.3). | “Really easily accessible… the information is very factual.” (P1) “To have it all in one place is a massive help… it’s trustworthy.” (P2)  “It looks very genuine because it’s so comprehensive.” (P3) “Keyword searches can be overwhelming… you go down a rabbit hole.” (P4)  “A nice little resource… something extra I can advise.” (P1) “Informative, easy to navigate, easy to understand.” (Survey) |
|  | 1.3 Increased confidence, mindset shifts and behavioural support | Captures ways the app shapes confidence, motivation, emotional management, and everyday behavioural choices. Includes reflections on self-help tools, mindset shifts, journalling, relational insight, and readiness for change. Does NOT include feelings of safety/support (1.4) or app usability (Theme 2). | “There’s a sense of security… motivation to address other things.” (P3)  “Made me more confident to engage in situations I wouldn’t have before.” (P3)  “I could actually do social things I would have put off.” (P3)  “It forced you to look at building and maintaining good relationships.” (P3)  “It does make a difference knowing I’ve got early intervention access.” (P4) “Journaling really helped me.” (P3)  “Information on unhelpful thinking styles… they found that helpful.” (P1)  “It probably hasn’t done anything I wouldn’t have done before.” (P4) “Increased my awareness of support and resources.” (Survey) |
|  | 1.4 Feeling supported, validated and empowered | Describes the app’s role as an ongoing companion that fosters emotional safety, reassurance, and validation. Includes the idea of a “safety net” between appointments. Does NOT include clinical alignment or factual trust (1.2), or behaviour change (1.3). | “It is to signpost… peace of mind for me as well.” (P2)  “You always need that security… you’re not going to pull out a leaflet.” (P3)  “A sense of security… it’s not going to disappear.” (P3)  “I recommended it because I’ll be on annual leave… it’s like safety netting.” (P1)  “A comfort blanket… having that resource.” (P4)  “It made me more positive… gives you a different outlook.” (P3)  “It provides me with a sense of security.” (Survey) |
| 2. Usability and engagement | 2.1 Clear layout and navigation | Focuses on user interface, layout, navigation, visual clarity, and structural organisation.  Does NOT include engagement with features (2.2) or content overwhelm (3.2). | “You can find crisis stuff in two buttons.” (P1)  “Clear, concise… flagged in red when important.” (P2)  “Very clearly laid out, even if distressed.” (P4)  “Easy to navigate… the subsections helped.” (P3)  “Icons people associate with those things.” (P4)  “Not very intuitive… like an ebook.” (Survey) |
|  | 2.2 Engagement and features | Describes how users interacted with features such as self-help tools, sections, and content variety. Includes reflections on what was engaging or lacking.  Does NOT include personalisation wishes (3.1). | “Apps are proving useful… especially for younger clients.” (P1)  “I check in… I know it’s always there.” (P3)  “Self-help stuff is particularly useful… books we use in clinic.” (P1)  “Lots of self-help ideas.” (P2) “A whole section for when you’re struggling.” (P4)  “Outdated user interface.” (P4) “Great for understanding red flags.” (Survey)  “Didn’t work to engage me.” (Survey) |
| 3. Improvements and concerns | 3.1 Personalisation and wider resources | Captures desires for personalised pathways, tailored content (e.g., neurodiversity), multimedia, local/national relevance, and built-in activities.  Does NOT include overwhelm (3.2). | “Wider resources are useful… but not national yet.” (P1)  “Seeing people in your own country… what’s available.” (P3) “Videos, podcasts… different ways to learn.” (P2)  “Resources for maintaining wellness… not just crisis.” (P4) “Interoception impairment, neurodiversity, ADHD…” (Survey)  “Provide audio description… different colour schemes.” (Survey)  “More downloadable resources… reflective activities.” (Survey) |
|  | 3.2 Information overwhelm and expectations management | Describes initial overload, complexity, and the need for clearer orientation or pacing. Includes discussions about expectations of what the app can/cannot do.  Does NOT include usability issues (Theme 2) unless specifically linked to overload. | “Initially overwhelmed… but later it felt positive and reassuring.” (P3)  “A very busy app… can be confusing.” (P4)  “Only harm is if I expected it to do everything.” (P3)  “Overwhelmed by information.” (Survey)  “Informative without being overwhelming.” (Survey) |
| 4. Inclusivity, language and tone | 4.1 Inclusivity and sensitivity | Focuses on the perceived inclusivity of diverse identities, ED presentations, life stages, and cultural groups.  Does NOT include tone (4.2). | “Sections on ethnic minorities, LGBTQI, dancers, pregnant people.” (P1)  “Everyone’s included… not just one population.” (P2) “  Tapped into disabilities… felt genuine.” (P3)  “Covers different cultures… would be great to hear from those groups.” (P4)  “Neurodiversity, ADHD, interoception impairment.” (Survey) |
|  | 4.2 Language clarity and non-judgemental tone | Captures perceptions of the tone as warm, clear, factual, non-stigmatising and easy to understand.  Does NOT include informational trust (1.2). | “Easy to understand… no jargon… not alienating.” (P3)  “Healthcare professionals can be dismissive… the app is not judgmental.” (P3)  “The tone is 100% what you would want… not condescending.” (P4)  “Neutral and informative.” (Survey)  “Professional, trusting but caring.” (Survey)  “Supportive and no judgment… inclusive and sensitive.” (Survey) |
